# Dihydroartemisinin Modulates Enteric Glial Cell Heterogeneity to Alleviate Colitis

**DOI:** 10.1002/advs.202403461

**Published:** 2024-07-11

**Authors:** Peishan Qiu, Ying Chang, Xiaoyu Chen, Shaoqi Wang, Haihang Nie, Yuntian Hong, Meng Zhang, Haizhou Wang, Cong Xiao, Yuhua Chen, Lan Liu, Qiu Zhao

**Affiliations:** ^1^ Department of Gastroenterology Zhongnan Hospital of Wuhan University Wuhan 430071 China; ^2^ Hubei Clinical Center & Key Lab of Intestinal & Colorectal Diseases Wuhan 430071 China

**Keywords:** colitis, dihydroartemisinin, dysbiosis, enteric glial cells, gut barrier, phenotypic transformation

## Abstract

The precise mechanism underlying the therapeutic effects of dihydroartemisinin (DHA) in alleviating colitis remains incompletely understood. A strong correlation existed between the elevation of glial fibrillary acidic protein (GFAP)^+^/S100 calcium binding protein B (S100β)^+^ enteric glial cells (EGCs) in inflamed colonic tissues and the disruption of the intestinal epithelial barrier (IEB) and gut vascular barrier (GVB) observed in chronic colitis. DHA demonstrated efficacy in restoring the functionality of the dual gut barrier while concurrently attenuating intestinal inflammation. Mechanistically, DHA inhibited the transformation of GFAP^+^ EGCs into GFAP^+^/S100β^+^ EGCs while promoting the differentiation of GFAP^+^/S100β^+^ EGCs back into GFAP^+^ EGCs. Furthermore, DHA induced apoptosis in GFAP^+^/S100β^+^ EGCs by inducing cell cycle arrest at the G0/G1 phase. The initial mechanism is further validated that DHA regulates EGC heterogeneity by improving dysbiosis in colitis. These findings underscore the multifaceted therapeutic potential of DHA in ameliorating colitis by improving dysbiosis, modulating EGC heterogeneity, and preserving gut barrier integrity, thus offering promising avenues for novel therapeutic strategies for inflammatory bowel diseases.

## Graphical abstract

In chronic colitis, microbial products or metabolites, along with inflammatory stimuli, can lead to concurrent damage to both the intestinal epithelial barrier (IEB) and the gut vascular barrier (GVB), accompanied by an increase in GFAP^+^/S100β^+^ enteric glial cells (EGCs) within the inflamed gut microenvironment. Dihydroartemisinin (DHA) regulates the heterogeneity of EGCs by improving dysbiosis, thereby effectively alleviating colitis and promoting the restoration of dual gut barrier function.

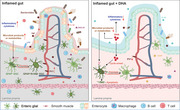

## 1 Introduction

Ulcerative colitis (UC) has traditionally been defined as diffuse, superficial inflammation that is restricted to the mucosa.^[^ ^1^, ^2^ ^]^ UC and Crohn's disease (CD) constitute the two primary types of inflammatory bowel disease (IBD), characterized as a group of chronic, relapsing, and inflammatory disorders.^[^ ^3^ ^]^ The impairment of gut barrier function and the presence of chronic inflammation serve as pivotal drivers of clinical complications in IBD. Consequently, the restoration of gut barrier integrity and mitigation of intestinal inflammation represent critical therapeutic objectives in the management of IBD.^[^ ^4^ ^]^ In 2015, Spadoni et al. put forward the concept of the gut vascular barrier (GVB), suggesting that the GVB and intestinal epithelial barrier (IEB) are distinct entities.^[^ ^5^ ^]^ Their proposition suggests that the GVB dismantling plays a crucial role in facilitating the entry of harmful substances, including toxins, into the systemic circulation, thereby leading to liver damage. The GVB controls the passage of antigens and commensal gut microbiota from the intestine into the bloodstream, preventing bacteria that have successfully crossed the IEB from entering the bloodstream.^[^ ^6^ ^]^ This insight underscores the potential benefits of enhancing dual‐barrier function as an underutilized strategy for treating IBD. While current research primarily focuses on the damage to the IEB during IBD, attention to the GVB offers a promising avenue for therapeutic intervention.

The enteric nervous system (ENS) is a “brain‐like” neural network that integrates several neurons and glia to give local control over vital gastrointestinal activities.^[^ ^7^ ^]^ Enteric glial cells (EGCs) are abundant peripheral glial cells that perform homeostatic functions in the gastrointestinal tract. Loss or enhancement of glial function can lead to alterations in the homeostasis of the ENS and gastrointestinal pathophysiology.^[^ ^7^ ^]^ In response to intestinal inflammation, enteric glia undergo dynamic changes both in vivo and in vitro, involving modifications in glial fibrillary acidic protein (GFAP) expression, morphological alterations, cytokine release, and shifts in gene expression profiles, as suggested by several studies.^[^ ^8^, ^9^, ^10^ ^]^ Despite these observations, the precise mechanisms underlying these responses remain elusive. Functioning as a central hub, EGCs regulate various processes such as homeostasis, pathogen defense, and tissue regeneration through direct and indirect interactions with different cell types within the gut.^[^ ^7^ ^]^ As proposed by Kabouridis et al. EGCs serve as primary targets for the gut microbiota, highlighting the regulatory role of microbes and their metabolites in the development and maturation of the EGC network.^[^ ^11^ ^]^ EGCs exhibit prominent heterogeneity and phenotypic plasticity, enabling them to adjust their functions based on triggers in the surrounding environment to preserve local homeostasis and facilitate effective resolution of intestinal inflammation. However, our understanding of the regional or local heterogeneity of EGCs and how their phenotypic changes affect gut barrier function and inflammation progression during enteritis remains limited. The potential relationship between microbiota diversity and EGC heterogeneity in intestinal inflammation remains poorly understood. In this study, we aimed to elucidate the relationship between alterations in EGC heterogeneity induced by chronic intestinal inflammation and changes in microbiota diversity. Furthermore, we sought to uncover the specific mechanisms by which these factors collectively regulate dual gut barrier function.

Artemisinin, derived from the herb Artemisia annua, is a sesquiterpene lactone peroxide. Its active metabolite, dihydroartemisinin (DHA), is extensively utilized for malaria treatment.^[^ ^12^ ^]^ Beyond its well‐known anti‐parasitic effects, recent studies have unveiled its diverse properties, including antiviral, antifungal, anticancer, and anti‐inflammatory activities.^[^ ^13^ ^]^ Notably, a study reported that DHA prominently inhibits microglial activation and neuroinflammation by modulating the miR‐16‐mediated Toll‐like receptor 4 (TLR4)/nuclear factor kappa B (NF‐κB) signaling pathway.^[^ ^7^ ^]^ Moreover, Gao et al. discovered that DHA ameliorates lipopolysaccharide (LPS)‐induced neuroinflammation by inhibiting the phosphatidylinositol 3‐kinase (PI3K)/protein kinase B (AKT) pathway, suggesting the potential of DHA in mitigating neuroinflammatory diseases.^[^ ^7^ ^]^ While the effects of DHA on the central nervous system (CNS) have been extensively investigated, its impact on the ENS remains less understood.^[^ ^14^ ^]^

We aimed to explore the pathogenesis and therapeutic strategies of colitis. Drawing insights from the GVB and the ENS, we delve into the essential drivers of gut barrier function and the role of EGCs in this process. Moreover, we focus on DHA as a potential treatment for UC, aiming to clarify its capacity to restore the dual gut barrier function and alleviate chronic colitis and its relationship with EGC heterogeneity and gut microbiota diversity.

## 2 Results

### 2.1 Dual Gut Barrier Impairment in Colitis

**Figure 1:**
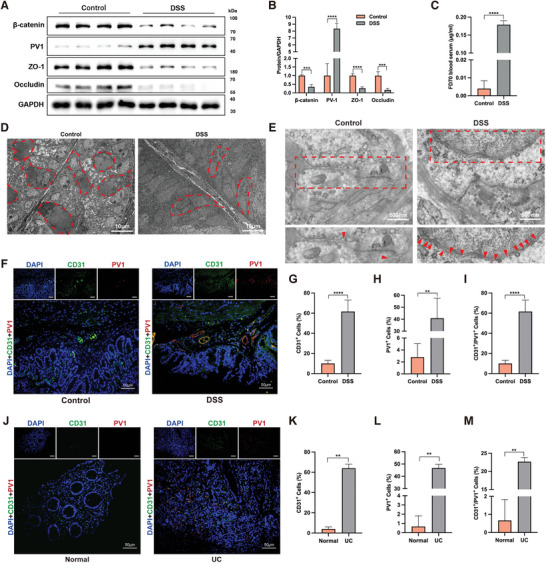
Dual Gut Barrier Impairment in Colitis. A,B) Western blot analysis of the level of IEB barrier proteins (ZO‐1 and Occludin) and GVB barrier proteins (β‐catenin and PV1) in the mucosa and lamina propria of control and chronic DSS‐induced colitis mice. C) Serum FITC‐dextran 70 kDa (FD70) concentration in control and chronic DSS‐induced colitis group. Representative images of goblet cells D) and tight junctions E) in colonic tissue from control and chronic DSS‐induced colitis mice by transmission electron microscopy (TEM). Red arrows indicated sites of tight junctions. F) Representative immunofluorescence images of PV1 (red) expression on colonic vessels stained with CD31 (green) in control and chronic DSS‐induced colitis mice. DAPI (blue) stained cell nuclei. Quantification of CD31^+^ cells G), PV1^+^ cells H), and CD31^+^/PV1^+^ cells I) in colon tissue from control and chronic DSS‐induced colitis mice. J) Representative immunofluorescence images of PV1 (red) expression on colonic vessels stained with CD31 (green) in healthy individuals and UC patients. DAPI (blue) stained cell nuclei. Quantification of CD31^+^ cells K), PV1^+^ cells L), and CD31^+^/PV1^+^ cells M) in colon tissue from healthy individuals and UC patients. About 50 cells were surveyed in each sample, and five fields of vision were used for quantification. IEB, intestinal epithelial barrier; GVB, gut vascular barrier; DSS, dextran sulfate sodium; DAPI, 4′,6′‐diamidino‐2‐phenylindole; UC, ulcerative colitis. Scale bars are shown in the figures. n = 5 for each group. Data are mean ± SEM. **p <0.01, ***p <0.001, ****p <0.0001.

To investigate the extent of gut barrier damage in inflamed colon tissues of colitis, considering that the damage primarily occurs in the mucosal and submucosal layers,^[^ ^15^, ^16^ ^]^ we obtained colon tissues from mice with dextran sulfate sodium (DSS)‐induced chronic colitis using a stripping method, which removed the muscular and serosal layers. Subsequently, we analyzed the expression of barrier proteins in the tissues (**Figure** **1**A,B). Our findings revealed that mice with chronic colitis exhibited impairment not only in the IEB (with downregulation of key tight junction (TJ) proteins zonula occludens‐1 (ZO‐1) and Occludin) but also in the GVB (downregulation of adherens junction (AJ) protein β‐catenin,^[^ ^5^ ^]^ upregulation of the plasmalemma vesicle‐associated protein 1 (PV1),^[^ ^17^ ^]^ a marker of vascular endothelial cell permeability^[^ ^18^, ^19^ ^]^). Furthermore, the damage to the GVB was evidenced by significantly elevated levels of LPS in the peripheral blood of colitic mice compared to the control group (Figure S1A, Supporting Information). The release of 70 kDa dextran from the bloodstream was observed (Figure 1C), as the shifting of molecules ≈70 kDa is known to be resisted by intact GVB.^[^ ^5^ ^]^ Simultaneously, the levels of inflammatory cytokines interleukin 1 beta (IL‐1β) and tumor necrosis factor (TNF) were significantly elevated in inflamed colon tissues compared to the control group (Figure S1B,C, Supporting Information), while the anti‐inflammatory cytokine interleukin 10 (IL‐10) showed a noticeable decrease in concentration (Figure S1D, Supporting Information). The electron microscopy results revealed a significant depletion of goblet cells in DSS‐induced colitis tissues (Figure 1D), along with an increase in fenestration of the endothelial cells at the tips of the villus in the colitis group (Figure 1E). Consistently, increased angiogenesis was observed in the mucosa, submucosa, and lamina propria of the colonic tissues of mice with colitis, accompanied by a significant upregulation of PV1 expression (Figure 1F–I). The histological examination of inflamed mucosal biopsies obtained from active UC patients and non‐IBD controls revealed extensive architectural tissue damage, characterized by disrupted epithelial barrier, significant loss of mucosal villi, disorganized glandular cell arrangement, prominent glandular loss, extensive infiltration of inflammatory cells, and reduced goblet cells (Figure S1E,F, Supporting Information). The histological scores for active UC patients were markedly elevated compared to the non‐IBD controls (Figure S1F, Supporting Information). Similar to findings in mice with colitis, inflamed mucosa from patients with UC demonstrated increased vascularization marked by platelet and endothelial cell adhesion molecule 1 (CD31)(Figure 1J,K), as well as a notable upregulation of PV1 expression (Figure 1J,L). It is noteworthy that the majority of newly formed blood vessels expressed high levels of PV1 (Figure 1M), indicating severe GVB damage in the inflamed biopsy specimens of patients with active UC. The above results indicate the presence of dual gut barrier damage, including the IEB and GVB, in the inflamed colon tissues of chronic colitic mice.

### 2.2 Increased Presence of GFAP^+^/S100 Calcium Binding Protein B (S100β)^+^ EGCs in Inflamed Colonic Tissues

**Figure 2:**
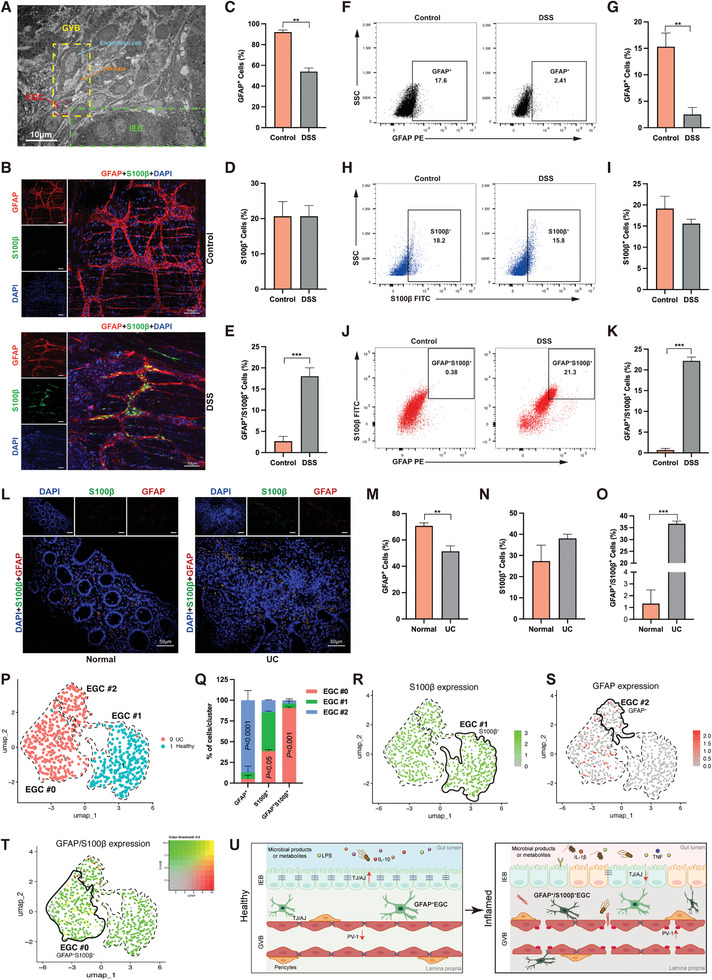
Increased abundance of GFAP^+^/S100β^+^ EGCs in inflamed colonic tissues. A) EGCs participate in gut vascular barrier (GVB) formation and are closely associated with intestinal epithelial barrier (IEB). Representative whole‐mount staining B) and quantification C–E) of GFAP (red) and S100β (green) in the submucosal plexus of control and chronic DSS‐induced colitis mice. DAPI (blue) stained the cell nuclei. The percentage of GFAP^+^ EGCs F,G) S100β^+^ EGCs H,I) and GFAP^+^/S100β^+^ EGCs J,K) were assayed in the colonic mucosa and lamina propria of control and DSS‐induced colitis mice. L) Representative immunofluorescence images of EGCs labeled with GFAP (red) and S100β (green) in full‐layer colonic sections of healthy individuals and UC patients. DAPI (blue) stained the cell nuclei. Quantification of GFAP^+^ cells M), S100β^+^ cells N), and GFAP^+^/S100β^+^ cells O) in colon tissue from healthy individuals and UC patients. P) Unsupervised subclustering of hEGC cluster 6 in healthy individuals (green dots) and those with UC (red dots). Q) Proportion of EGCs expressing GFAP or S100β in each subpopulation. Single cells possessing greater than 3 and non‐zero values are defined as GFAP^+^ and S100β^+^, respectively (n = 5 Drop‐seq replicates). Unsupervised analysis of EGC clusters shows that EGC subpopulation gene signatures correlate with S100β R), GFAP S), and S100β+GFAP T) enrichment in EGC#1, EGC#2, and EGC#0, respectively. U) Schematic representation of the mucosal and submucosal microenvironment in the gut under healthy and inflamed conditions: In the healthy state, GFAP^+^ EGCs predominate, while in the inflamed state, there is an increase in GFAP^+^/S100β^+^ EGCs. About 50 cells were surveyed in each sample, and five fields of vision were used for quantification. DSS, dextran sulfate sodium; EGCs, enteric glial cells; DAPI, 4′,6′‐diamidino‐2‐phenylindole; UC, ulcerative colitis. Scale bars are shown in the figures. *n* = 5 for each group. Data are mean ± SEM. **p <0.01, ***p <0.001.

As depicted by the ultrastructure of DSS‐induced colitis mouse colon tissues, gut microvascular endothelial cells are surrounded by EGCs and pericytes, collectively forming the intact gut‐vascular unit^[^ ^5^ ^]^ (**Figure** **2**A). The IEB is made up of a monolayer of intestinal epithelial cells structured into invaginations (crypts) and finger‐like projections (villi) in the small intestine, as well as a series of crypts alternating with a flat epithelial surface in the colon.^[^ ^20^ ^]^ EGCs anatomically reside within the IEB/GVB (Figure 2A). Given the pivotal anatomical location of EGCs, involved in both the formation of GVB and their association with the IEB, we further assessed the expression of EGCs in the inflamed colon full‐layer sections of mice with DSS‐induced colitis. Regarding DSS‐induced inflammation, S100β^+^ cells constituted the great majority of GFAP^+^ cells, representing our primary identification of the EGCs subtype (Figure S2A, Supporting Information). Previous evidence indicates continuous dynamic regulation of gene and protein expression in subtypes of enteric glia, alongside potential variations in responsiveness to intercellular mediators both within and across these subtypes.^[^ ^21^, ^22^ ^]^ Moreover, it is evident that GFAP^+^/S100β^+^ EGCs in colitic tissues predominantly localize within the mucosa and submucosa (i.e., the areas affected by inflammation). The identification of elevated GFAP and S100β protein expression in the inflamed colonic tissues after the removal of the serosa and myenteron provided further evidence in support of this finding (Figure S2B,C, Supporting Information). To reinforce this finding, we performed submucosal whole‐mount IF staining (Figure 2B–E). Our analysis revealed a modest reduction in GFAP^+^ EGCs in the submucosa of colitic groups compared to controls, while no notable disparity in S100β^+^ EGCs was observed between the cohorts. Additionally, the GFAP^+^/S100β^+^ EGCs exhibited a significant upregulation in the submucosa of mice with DSS‐induced colitis.

To furnish direct evidence of increased expression of GFAP^+^/S100β^+^ EGCs in the inflamed colonic mucosa and submucosa and to gain further insights into downstream signaling events, we isolated primary EGCs from the inflamed colonic mucosa and submucosa of DSS‐induced colitis mice. Subsequently, we utilized flow cytometry to assess the expression of marker proteins (Figure 2F–K). Consistent with the observations of submucosal immunofluorescence, although no noticeable distinction was observed in S100β^+^ EGCs across the groups, there was a reduction in GFAP^+^ EGCs. Conversely, there was a notable increase in GFAP^+^/S100β^+^ EGCs within the submucosa of the colitic groups compared to the control groups. Crucially, in the inflamed biopsy tissues of patients with active UC, GFAP^+^/S100β^+^ EGCs were predominantly found among areas with inflammatory injury (Figure 2L,O). The findings observed in patients with active UC are consistent with those observed in mice with DSS‐induced colitis, where GFAP^+^ EGCs were slightly decreased in inflamed intestinal tissues compared to normal controls (Figure 2L,M), and the expression of S100β^+^ EGCs showed no significant difference between the two groups (Figure 2L,N). This further supports the notion of increased expression of GFAP^+^/S100β^+^ EGCs in inflamed colonic tissues.

To explore the heterogeneity of EGC in colitis tissues, we analyzed single‐cell RNA sequencing (scRNA‐seq) data from colonic mesenchyme cell populations of healthy controls and patients with UC. After doing an unsupervised clustering analysis, we identified 12 disparate stromal cell populations (Figure S2H, Supporting Information). Utilizing glial cell markers S100β and GFAP, we designated cluster 6 as the EGC population. Subsequently, we defined three subpopulations inside the EGC cluster 6 using unsupervised sub‐clustering analysis, denoted as EGC#0–EGC#2 (Figure S2I, Supporting Information). As shown in Figure 2P, we discovered that whereas EGC#0 and EGC#2 are more common in patients with UC, cluster EGC#1 is more prevalent in healthy material. Upon comparing the expression of S100β and GFAP, within these subclusters, we discovered that while S100β was expressed to variable degrees within most subpopulations, EGC#1 exhibited consistently high S100β expression, with 48.5% of S100β^+^ cells (Figure S2K, Supporting Information; Figure 2Q,R), in contrast to 36.4% and 15% in EGC#0 and EGC#2, respectively. On the other hand, EGC#2 had 77% of GFAP^+^ cells, whereas EGC#0 and EGC#1 had 7.7% and 15.3%, respectively (Figure S2J, Supporting Information; Figure 2Q,S). Furthermore, GFAP expression was higher in EGC#2, whereas S100β was substantially expressed in EGC#1. Most notably, we observed that GFAP^+^/S100β^+^ EGCs were predominantly enriched in cluster 0, comprised almost entirely of EGCs sourced from UC patients (Figure 2Q,T, highlighted in yellow). Delineating GFAP^+^/S100β^+^, S100β^+^, and GFAP^+^ labels for EGC#0, EGC#1, and EGC#2, respectively, this scRNA‐seq study highlights the variability of EGC populations. The evidence above substantiates the assertion that multiple phenotypes of EGCs exist within colonic tissues, with a conspicuous increase in GFAP^+^/S100β^+^ EGCs observed in the inflamed colon (Figure 2U).

### 2.3 The Protective Role of DHA in Colitis – Restoring Dual Gut Barrier Function

**Figure 3:**
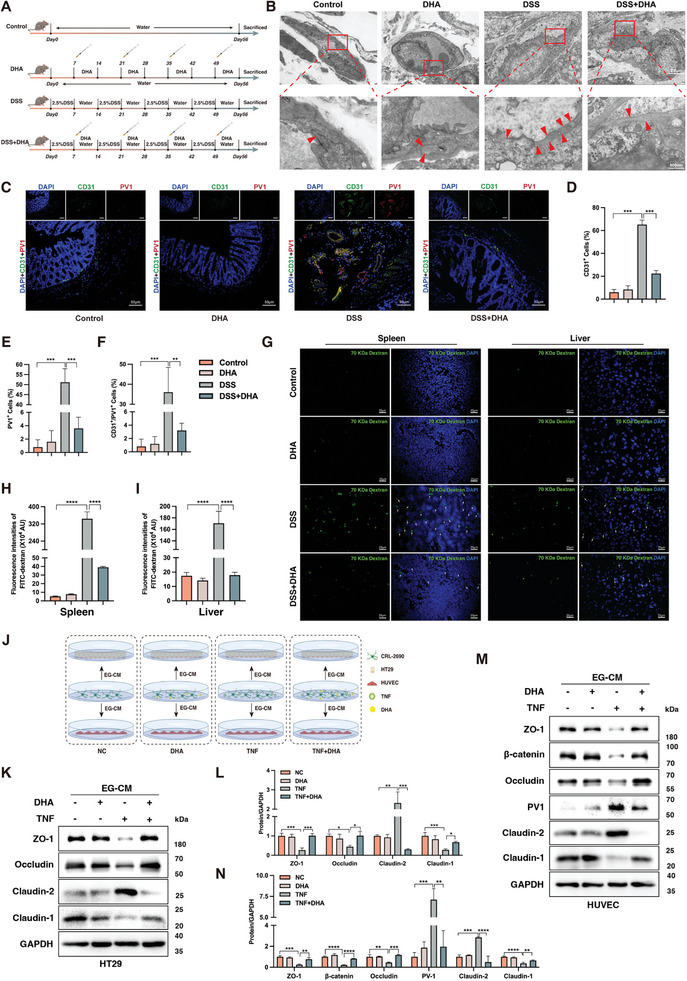
DHA protects dual gut barrier function in colitis. A) Flowchart of animal experiment modeling process. B) Representative images of tight junctions in colonic tissue from each group of mice by transmission electron microscopy (TEM). Red arrows indicated sites of tight junctions. C) Representative immunofluorescence images of PV1 (red) expression on colonic vessels stained with CD31 (green) in each group. DAPI (blue) stained cell nuclei. Quantification of CD31^+^ cells D), PV1^+^ cells E), and CD31^+^/PV1^+^ cells F) in colon tissue from each group. About 50 cells were surveyed in each sample, and five fields of vision were used for quantification. G) The integrity of the GVB was assessed by measuring the fluorescence intensity of FD70 (green), referring to the emergence of FD70 in the liver and spleen after gut lumen exposure to FD70 in different groups. Quantification of fluorescence intensity of FD70 in the spleen H) and liver I) from each group. J) Experimental illustration of DHA's impact on IEB/GVB via EGCs modulation under inflammatory conditions. K‐L) Western blot analysis of the level of IEB barrier proteins (ZO‐1, Occludin, Claudin‐2, and Claudin‐1) in HT29 under EGCs conditioned medium treatment with or without DHA therapy under inflammatory conditions. M‐N) Western blot analysis of the level of GVB barrier proteins (ZO‐1, β‐catenin, Occludin, PV1, Claudin‐2, and Claudin‐1) in HUVECs under EGCs conditioned medium treatment with or without DHA therapy under inflammatory conditions. DHA, dihydroartemisinin; DSS, dextran sulfate sodium; DAPI, 4′,6′‐diamidino‐2‐phenylindole; FD70, FITC‐labeled fluorescent dextran 70 KDa; IEB, intestinal epithelial barrier; GVB, gut vascular barrier; EG‐CM, enteric glial‐conditioned medium; EGCs, enteric glial cells. Scale bars are shown in the figures. n = 5 for each group (B, C, and G). Data are mean ± SEM. *p < 0.05, **p < 0.01, ***p < 0.001, ****p < 0.0001.

To explore the anti‐inflammatory effects of DHA in chronic colitis, we administered intraperitoneal injections of 20 mg kg^−1^ (body weight) of DHA daily to DSS‐treated C57BL/6 mice (**Figure** **3**A). After one week of continuous administration, the treatment was paused for one week before resuming, repeating four rounds to conclude the modeling. During DSS administration, the DHA‐treated group showed significantly reduced body weight loss compared to the colitic mice (Figure S3A, Supporting Information), manifested as body weight loss appearing earlier and being more severe at any time point in DSS‐induced mice not treated with DHA. At the conclusion of the experiment, the improvement in colon length shortening (Figure S3B, Supporting Information) observed in the DHA‐treated group confirmed a significant difference in disease activity scores (Figure S3C, Supporting Information) and spleen index (Figure S3D, Supporting Information) between the groups. Histological examination of colon cross‐sections revealed that mice in the DHA‐treated group exhibited smaller areas of colonic inflammation, improved tissue edema, and less disrupted crypt architecture compared to the DSS group, with contractible immune cell infiltration and lower colonic histological scores (Figure S3E,F, Supporting Information). In addition, our real‐time quantitative polymerase chain reaction (qPCR) results (Figure S3G, Supporting Information) revealed that compared to colitis mice untreated with DHA, those in the DHA‐treated group exhibited significantly reduced expression of inflammatory cytokines, such as interleukin 6 (*IL6)*, *IL1B*, interferon gamma (*IFNG)*, and *TNF*. The expression of the anti‐inflammatory cytokine *IL10* was augmented following DHA administration. Mice colon cross‐sections in steady state (no DSS) showed no signs of pathology. Furthermore, DHA treatment markedly decreased the levels of inflammatory cytokines IL‐1β (Figure S3H, Supporting Information) and TNF (Figure S3I, Supporting Information) in inflamed colonic tissues, while elevating the content of the anti‐inflammatory cytokine IL‐10 (Figure S3J, Supporting Information). Additionally, DHA supplementation also attenuated the elevation of LPS levels in the peripheral blood of DSS‐induced colitis mice (Figure S3K, Supporting Information), providing more extensive evidence for the protective role of DHA in the context of colitis.

To determine whether DHA can improve the dual gut barrier function in DSS‐induced colitis, we performed the western blot analysis on colonic tissues from mice with mucosal and muscular layers removed (Figure S3L,M, Supporting Information). As predicted, our results indicated that DHA administration significantly restored the downregulation of markers related to IEB integrity induced by DSS, such as the TJ proteins (ZO‐1 and Occludin). More importantly, the AJ protein β‐catenin associated with GVB was elevated following DHA treatment, while the expression of GVB permeability‐related molecule PV1 was reduced by DHA administration. To further investigate the reparative effect of DHA on GVB in colitis, the ultrastructure of GVB in the colonic tissues of each group of mice was examined using transmission electron microscopy (TEM), as shown in Figure 3B. Moreover, we observed through immunofluorescence staining that the colonic whole‐layer sections of mice treated with DHA (Figure 3C) exhibited a more pronounced reduction in CD31^+^/PV^+^ cells compared to colitis mice with no DHA treated (Figure 3F). The increase in CD31^+^ cells (Figure 3D) and PV1^+^ cells (Figure 3E) in the inflamed colons of DSS‐induced mice was also blocked by DHA treatment, indicating that DHA can inhibit neovascularization in colitis mice, especially the generation of highly permeable endothelial cells. The results revealed a significant reduction in the fenestration of vascular endothelial cells in the colonic tissues of mice treated with DHA compared to those without DHA treatment.

We further evaluated the effect of DHA treatment on gut barrier function in colitis by conducting fluorescein isothiocyanate (FITC)‐dextran intestinal permeability assay in each group of mice. This experiment serves as the most direct assessment of GVB permeability.^[^ ^23^ ^]^ In line with TEM observations, DHA administration significantly reduced the levels of FITC‐dextran in the serum of colitis mice (Figure S3N, Supporting Information). Indeed, the frozen sections of the liver and spleen from mice treated with DHA exhibited significantly attenuated green fluorescence intensity under the microscope as compared with colitis mice not treated with DHA (Figure 3G–I), providing evidence for the extensive role of DHA in mediating dual gut barrier protection in colitis. Given that EGCs are positioned at the critical anatomical site associated with IEB/GVB (Figure 2A), and the occurrence of the abnormally increased proportion of GFAP^+^/S100β^+^ EGCs in inflamed colonic tissues correlates positively with the presence of dual gut barrier damage, more importantly, both are significantly improved after DHA treatment. These findings suggest a close association between DHA's reparative effect on dual gut barrier damage during enteritis and EGCs. To further elucidate the impact of DHA on dual gut barrier function through its action on EGCs in the context of inflammation, we incubated EGCs with DHA in vitro and obtained the conditioned medium. Subsequently, we treated intestinal epithelial cells (HT29) and vascular endothelial cells (HUVECs) with the conditioned medium separately and assessed the expression of relevant barrier proteins (Figure 3J). Regarding the impact of DHA‐treated TNF‐activated EGCs on IEB, compared to the untreated inflammation group, western blot analysis revealed an upregulation in the expression of TJ proteins ZO‐1, Occludin, and Claudin‐1, along with a downregulation in Claudin‐2 (a modulator of intestinal inflammation‐related leaky gut barrier)^[^ ^24^ ^]^ (Figure 3K,L). These findings demonstrated the reparative effect of DHA‐treated TNF‐activated EGCs on IEB. Consistently, the impact of DHA‐treated TNF‐activated EGCs on GVB was also favorable. Compared to the inflammation group without DHA treatment, similar trends were observed in the expression of TJ proteins within the GVB as observed in the IEB (Figure 3M,N). Additionally, the expression of AJ‐associated protein β‐catenin in GVB was upregulated, along with a decrease in the fenestration protein PV1 in the DHA‐treated inflammation group. These results indicated that DHA administration not only restores the integrity of IEB but also repairs the GVB.

### 2.4 Modulation of EGC Phenotypes by DHA in Colitis

**Figure 4:**
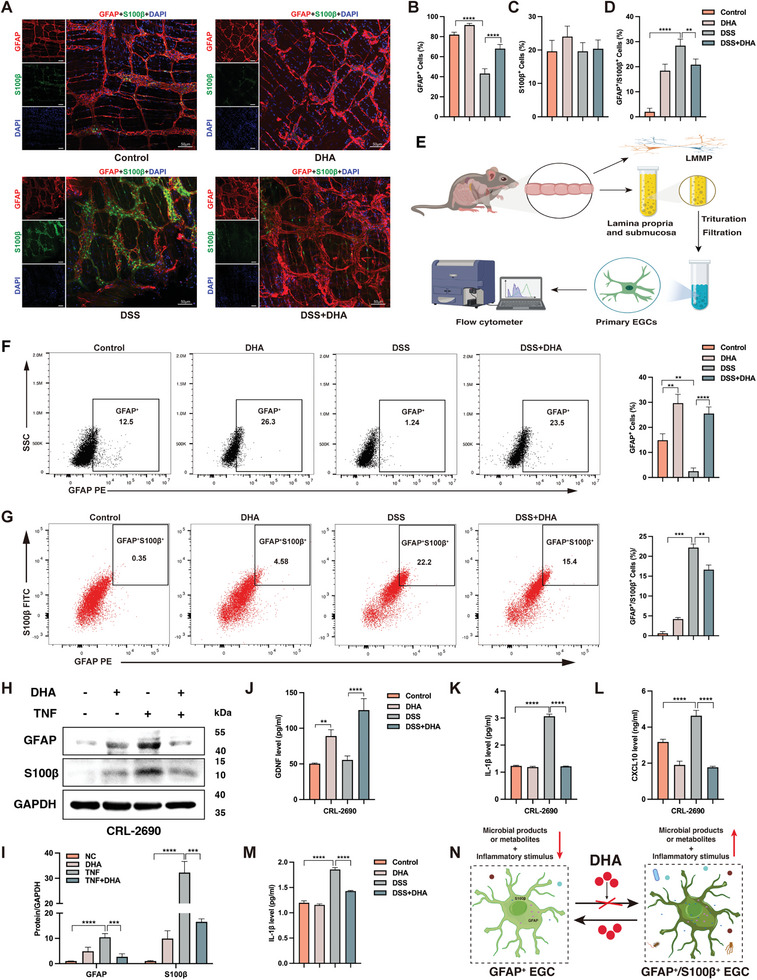
DHA regulation of EGC heterogeneity. A) Representative whole‐mount staining and quantification B–D) of GFAP (red) and S100β (green) in the submucosal plexus of different groups of mice. DAPI (blue) stained the cell nuclei. Quantification of GFAP^+^ EGCs (B), S100β^+^ EGCs (C), and GFAP^+^/S100β^+^ EGCs (D) in the submucosal plexus of different groups of mice. E) Schematic diagram of mouse colonic primary EGCs extraction for flow cytometry analysis. The percentage of GFAP^+^ EGCs F) and GFAP^+^/S100β^+^ EGCs G) were assayed in the colonic mucosa and lamina propria of colitis mice treated with or without DHA and corresponding control mice. H‐I) Western blot analysis of the expression of EGCs surface markers (GFAP and S100β) under inflammatory conditions with or without DHA treatment. The intracellular levels of GDNF J), IL‐1β K), and CXCL10 L) in EGCs of each group determined by ELISA. M) The extracellular IL‐1β levels in EGCs of each group determined by ELISA. N) Graphical abstract of the modulation of EGC heterogeneity by DHA under inflammatory conditions: DHA inhibits the transformation of GFAP^+^ EGCs into GFAP^+^/S100β^+^ EGCs and promotes the conversion of GFAP^+^/S100β^+^ EGCs into GFAP^+^ EGCs in the context of inflammation. DHA, dihydroartemisinin; DSS, dextran sulfate sodium; LMMP, longitudinal muscle layer, and the longitudinal muscle/myenteric plexus; EGCs, enteric glial cells; DAPI, 4′,6′‐diamidino‐2‐phenylindole. Scale bars are shown in the figures. n = 5 for each group (A, F, and G). Data are mean ± SEM. **p < 0.01, ***p < 0.001, ****p < 0.0001.

To further dissect the protective role of DHA in experimental colitis in vivo, the western blot analysis of colonic tissues, depleted of the serosa and myenteron, indicated a dramatic decrease in the expression of GFAP and S100β protein in the DHA‐treated group compared to the untreated group of colitis mice (Figure S4A,B, Supporting Information), reinforcing the beneficial role of DHA in DSS‐induced colitis. Immunofluorescence staining of colonic cross‐sections (Figure S4C, Supporting Information) also exhibited a similar trend, wherein the fluorescence intensity of GFAP and S100β in the colitis mice treated with DHA was weaker compared to the untreated group. Furthermore, we observed a more obvious augment in the number of GFAP^+^ cells in the DHA‐treated group compared to those untreated with DHA but with colitis (Figure 4B), as indicated by immunofluorescence analysis of submucosal whole‐mount IF staining (**Figure** **4**A). Interestingly, the treatment with DHA showed no significant effect on the abundance of S100β^+^ cells (Figure 4C). Of particular note, DHA treatment significantly suppressed the content of GFAP^+^/S100β^+^ cells in DSS‐induced colitis (Figure 4D), providing further substantiation for the broader pivotal role of DHA in managing gut inflammation. Given the crucial role of GFAP^+^ EGCs in the repair of IBD,^[^ ^25^ ^]^ we thus hypothesize that in the inflammatory context, DHA not only inhibits the differentiation of GFAP^+^ EGCs into GFAP^+^/S100β^+^ EGCs but also promotes the transformation of GFAP^+^/S100β^+^ EGCs to GFAP^+^ EGCs.

To investigate the exact relationship between DHA inhibition of GFAP^+^/S100β^+^ EGCs phenotypic transformation in inflamed colonic tissues and colitis remission, we extracted primary EGCs from colonic tissues of each group of mice according to the protocol provided by Wang et al.^[^ ^26^ ^]^ We then conducted flow cytometry to assess their phenotypic changes (Figure 4E). In DSS‐induced colitis, flow cytometry analysis revealed that 1.24% of EGCs were GFAP^+^, while in the colitis group treated with DHA, the proportion of GFAP^+^ EGCs increased to 23.5% (Figure 4F). Consistent with previous observations, DSS did not induce an increase in the proportion of S100β^+^ cells in colitis mice. However, treatment with DHA induced this property, especially in the colitis group, as the ratio of S100β^+^ cells in mice treated with DHA was more than doubled compared to the untreated group (Figure S4D, Supporting Information). It is worth noting that the significant increase in GFAP^+^/S100β^+^ EGCs observed in the DSS‐induced colitis group was markedly reduced after DHA administration (Figure 4G), reinforcing the hypothesis that DHA can induce the transition of GFAP^+^/S100β^+^ EGCs to GFAP^+^ EGCs in the inflamed colon. Next, we sought to determine whether the EGC‐specific changes observed in vivo could also be replicated in vitro. We pre‐incubated EGC line CRL‐2690 with TNF for 24 h to simulate the inflammatory damage caused by colitis (Figure 4H). Subsequently, Western blot analysis revealed that DHA treatment reduced the elevated protein levels of GFAP and S100β following TNF pre‐incubation (Figure 4I), providing decisive evidence for the fact that DHA can convert the increased GFAP^+^/S100β^+^ EGCs observed in DSS‐induced colitis to GFAP^+^ EGCs.

Reactive EGC transcriptome profiling in humans and mice indicates that EGCs enhance the generation of neurotrophin (like glial cell‐derived neurotrophic factor (GDNF)), inflammatory cytokines (like IL‐1β), and chemokines (like C‐X‐C motif chemokine ligand 10 (CXCL10)).^[^ ^8^, ^9^ ^]^ Furthermore, we found that regardless of inflammation, DHA treatment significantly increased the intracellular levels of the barrier‐enhancing factor GDNF in EGCs (Figure 4J), as reported previously,^[^ ^27^ ^]^ without affecting the secretion of GDNF by EGCs (Figure S4E, Supporting Information). This suggests that the barrier‐protective effect exerted by EGC through GDNF is not mediated via secretion. Inflammation led to a significant increase in the intracellular levels of IL‐1β (Figure 4K) and CXCL10 (Figure 4L) in EGCs. However, treatment with DHA markedly reduced their intracellular levels in EGCs. Even more importantly, DHA alleviates the increase in IL‐1β secretion induced by inflammation in EGCs (Figure 4M). Pre‐treatment with TNF significantly reduces the ability of EGCs to secrete CXCL10, indicating that inflammation impairs the chemotactic ability of EGCs toward immune cells (Figure S4F, Supporting Information). However, DHA treatment does not increase the secretion of CXCL10 by EGCs. Building on the previous findings (Figure 3), this also underscores the close association between the functional damage of IEB/GVB and GFAP^+^/S100β^+^ EGCs. These studies demonstrate that DHA possesses the ability to promote the phenotypic transition of increased GFAP^+^/S100β^+^ EGCs to GFAP^+^ EGCs during intestinal inflammation while simultaneously inhibiting the differentiation of GFAP^+^ EGCs into GFAP^+^/S100β^+^ EGCs (Figure 4N).

### 2.5 DHA‐Induced Cell Cycle Arrest and Apoptosis of GFAP^+^/S100β^+^ EGCs in the Inflammatory State

**Figure 5:**
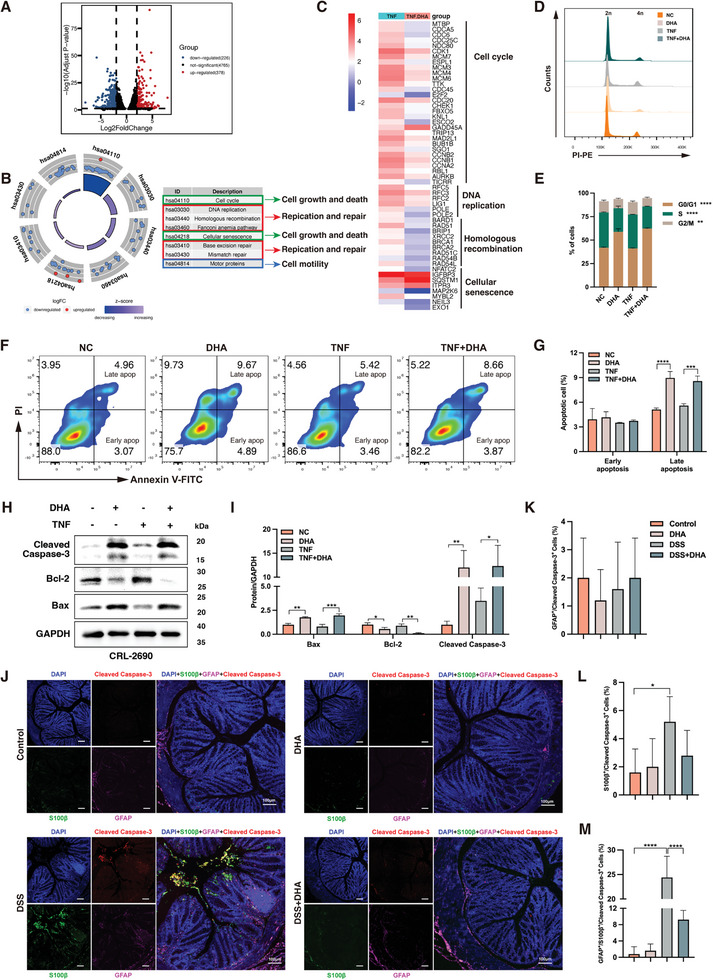
In the inflammatory condition, DHA causes GFAP^+^/S100β^+^ EGCs to enter a cell cycle arrest and undergo apoptosis. A) Volcano plot of actively transcribed genes in inflamed EGCs after DHA treatment, with significantly differentially expressed genes (DEGs) (*P*‐value < 0.05,> ± twofold) marked red (upregulated) and blue (downregulated). B) KEGG pathway enrichment analysis of DEGs in inflamed EGCs after DHA treatment. C) Heatmap of partial DEGs for KEGG enrichment analysis. D) Cell cycle analysis of inflammatory EGCs with or without DHA treatment. E) Quantitative analysis of cell cycle. F) Cell apoptosis analysis of inflammatory EGCs with or without DHA treatment. G) Quantitative analysis of cell apoptosis. H‐I) Western blot analysis of apoptotic protein (Cleaved Caspase‐3, Bcl‐2, and Bax) expression in EGCs treated with or without DHA under inflammatory conditions. J) Representative immunofluorescence images of Cleaved Caspase‐3 (red) expression on EGCs stained with GFAP (magenta) and S100β (green) in full‐layer colonic sections of colitis mice treated with or without DHA and corresponding control mice. DAPI (blue) stained cell nuclei. Quantification of GFAP^+^/Cleaved Caspase‐3^+^ cells (K), S100β^+^/Cleaved Caspase‐3^+^ cells L), and GFAP^+^/S100β^+^/Cleaved Caspase‐3^+^ cells M) in colon tissue from each group. About 50 cells were surveyed in each sample, and five fields of vision were used for quantification. DHA, dihydroartemisinin; DSS, dextran sulfate sodium; EGCs, enteric glial cells; DAPI, 4′,6′‐diamidino‐2‐phenylindole; KEGG, Kyoto Encyclopedia of Genes and Genomes. Scale bars are shown in the figures. n = 5 for each group J). Data are mean ± SEM. *p < 0.05, **p < 0.01, ***p < 0.001, ****p < 0.0001.

To investigate the molecular mechanisms underlying the effect of DHA on intestinal inflammation, we conducted RNA‐sequencing (RNA‐Seq) transcriptomic analysis on EGCs treated with and without DHA following TNF pre‐incubation. Volcano plots comparing DHA treated with untreated inflammatory samples showed 378 up‐regulated genes and 226 down‐regulated genes (**Figure** **5**A). We subsequently performed Gene Ontology (GO) analysis on these differentially expressed genes (DEGs) to elucidate the biological processes (BP), cellular components (CC), and molecular functions (MF) they are engaged in, aiming to further investigate the impact of DHA treatment on TNF pre‐incubated EGCs (Figure S5A, Supporting Information). It revealed that compared to the untreated inflammatory EGCs, the DHA‐treated inflammatory EGCs showed enrichment in BP related to chromosome segregation, CC associated with the chromosomal region, and MF related to adenosine triphosphate (ATP) and DNA‐related activities. This indicates that DHA treatment mainly affects the cell division and proliferation of inflammatory EGCs. The Kyoto Encyclopedia of Genes and Genomes (KEGG) analysis of DEGs revealed that the enriched pathways primarily involve cell cycle, DNA replication, cellular senescence, homologous recombination, base excision repair, and mismatch repair‐related KEGG pathways (Figure 5B). Moreover, these pathways mainly belong to the KEGG categories of cellular growth and death (hsa04110 and hsa04218), replication and repair (hsa03030, hsa03440, hsa03460, hsa03410, and hsa03430). The heatmap in Figure 5C illustrates the DEGs associated with cell cycle, DNA replication, cellular senescence, and homologous recombination. The RNA‐Seq data revealed that DHA treatment had the most significant impact on the cell cycle of EGCs pre‐incubated with TNF. Consequently, we assessed the cell cycle changes in EGCs from each group (Figure 5D). We observed a dramatic increase in the proportion of cells in the G0/G1 phase and a corresponding decrease in cells in the S and G2/M phases in EGCs after DHA treatment, suggesting that DHA can arrest the cell cycle of EGCs at the G0/G1 phase (Figure 5E), further indicating that the effect of DHA on inflammatory EGCs involves proliferation inhibition.

Further expanding our current RNA‐Seq study and complementing it with flow cytometry analysis, we can confirm no statistically significant differences in the early apoptosis rates among the cell groups (Figure 5F,G). However, DHA treatment significantly increased the late apoptosis rate of EGCs, regardless of whether the EGCs were pre‐treated with TNF, suggesting a lethal role of DHA in EGCs. Moreover, we detected the expression of apoptosis‐related proteins in EGCs pre‐treated with TNF and then treated with DHA. As anticipated, DHA treatment significantly upregulated the expression of Bax and Cleaved Caspase‐3 in inflamed EGCs and downregulating the expression of Bcl‐2, supporting the notion that DHA can induce apoptosis in GFAP^+^/S100β^+^ EGCs (Figure 5H,I). To further investigate the apoptosis of GFAP^+^/S100β^+^ EGCs in vivo, we detect the expression of the well‐known apoptosis protein Cleaved Caspase‐3^[^ ^28^ ^]^ by immunofluorescence in healthy control and UC patients’ colon biopsy (Figure S5B–E, Supporting Information). Consistent with the observations in the in vitro inflammatory‐induced EGCs model, we found no significant difference in the apoptosis rates of GFAP^+^ EGCs (Figure S5C, Supporting Information) and S100β^+^ EGCs (Figure S5D, Supporting Information) in the inflamed colon of UC patients compared to healthy controls. However, the UC group indicated an overall increased number of apoptotic GFAP^+^/S100β^+^ EGCs (Figure S5E, Supporting Information). In another set of experiments, we aimed to investigate the effect of DHA on inducing apoptosis of GFAP^+^/S100β^+^ EGCs in vivo (Figure 5J–M). There was no statistically significant apoptosis observed in GFAP^+^ EGCs among the groups (Figure 5K). We found that both S100β^+^ EGCs (Figure 5L) and GFAP^+^/S100β^+^ EGCs (Figure 5M) exhibited increased apoptosis in DSS‐induced colitis mice. However, only GFAP^+^/S100β^+^ EGCs showed a significant decrease in apoptosis rate after DHA treatment. Taken together, these findings demonstrate that DHA induces cell cycle arrest and apoptosis in EGCs, particularly in GFAP^+^/S100β^+^ EGCs.

### 2.6 DHA Modulated EGC Heterogeneity by Regulating Gut Dysbiosis in DSS‐Induced Colitis

Previous studies,^[^ ^21^, ^22^, ^29^, ^30^ ^]^ in conjunction with our aforementioned findings, reveal dynamic changes in the expression levels of major glial markers in EGCs, influenced by environmental factors and stimuli encountered by EGCs. Moreover, the gut microbiota profoundly impacts the abundance of EGCs within the gut.^[^ ^11^, ^31^ ^]^ Kabouridis et al. definitively demonstrated that EGCs are the primary target cells of the gut microbiota, and in‐situ gut microbiota regulates the homeostasis of EGCs within the intestinal mucosa.^[^ ^11^ ^]^ Additionally, the expression of GFAP, which is observable, varies depending on the neuroglial state,^[^ ^8^ ^]^ subtype,^[^ ^21^ ^]^ and genetic targeting strategies.^[^ ^32^ ^]^ Spica et al. suggested that glial‐derived S100β is involved in mediating the regulation of gut microbiota diversity.^[^ ^33^ ^]^ Thus, to investigate whether DHA treatment alters the gut microbiota of colitis mice and consequently affects EGC heterogeneity, we evaluated the intestinal microbiota diversity in mouse feces.

**Figure 6:**
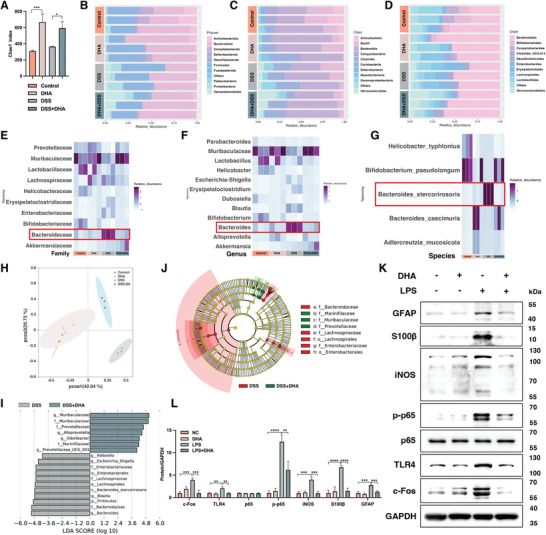
16S ribosomal RNA (rRNA) sequencing analysis of gut microbiota of colitis mice treated with or without DHA and corresponding control mice. A) Chao1 index of each group showed the 𝛼‐diversity of the microbial community. The community histogram showed the microbial compositional profiling at the phylum B), class C), and order D) levels. Each row represents each mouse and n = 3 for each group. Heatmap exhibited the relative abundance of microbial compositional profiling at the family E), genus F), and species G) levels. Each column represents each mouse and n = 3 for each group. H) A principal co‐ordinates analysis revealed the gut microbiome's diversity. Each point represents each mouse and n = 3 for each group. Clustering's significance was assessed using analysis of similarities (ANOSIM). I) Linear discriminant analysis (LDA) determines the most abundant genus in various groupings. Taxa that meeting an LDA significant threshold of 3.6 are shown. J) A cladogram revealed the disparity in richness, as well as the group with the greatest abundance. The brightness of each dot positively relates to its effect. K‐L) Western blot analysis of the expression of GFAP, S100β, and bacterial product‐related signaling molecules (c‐Fos, TLR4, NF‐κB, and iNOS) of EGCs after pre‐incubation with LPS with or without DHA treatment. DHA, dihydroartemisinin; DSS, dextran sulfate sodium; EGCs, enteric glial cells; LPS, lipopolysaccharide. Data are mean ± SEM. *p < 0.05, **p < 0.01, ***p < 0.001, ****p < 0.0001.

Results from 16S ribosomal RNA (rRNA) gene sequencing of all four groups (Control, DHA, DSS, and DSS+DHA) of mice showed that the richness and diversity of microbial communities, as indicated by the Chao index (**Figure** **6**A), were significantly increased after DHA treatment, regardless of the occurrence of colitis. Furthermore, compared to normal control mice, at the phylum level, there was a significant decrease in the proportions of Actinobacteriota and Campylobacterota but a significant increase in Proteobacteria in the feces of colitis mice (Figure 6B). At the class level, Actinobacteria was significantly reduced in colitis mice, while Gammaproteobacteria showed a significant increase. Treatment with DHA helped improve this situation (Figure 6C). At the order level, Bifidobacteriales showed an observable decrease in the feces of colitis mice, but DHA administration did not improve this condition (Figure 6D). At the family level, there was a notable increase in the abundance of Bacteroidaceae in the colitis group compared to the control group. However, in the colitis group treated with DHA, the proportion of Bacteroidaceae decreased (Figure 6E). Similarly, Bacteroides at the genus level showed a significant upregulation in the DSS‐induced colitis group, which was markedly attenuated by DHA treatment (Figure 6F). Furthermore, at the species level, the significantly elevated proportion of Bacteroides_stercorirosoris in the colitis group decreased after DHA administration (Figure 6G). These findings collectively suggest that the alterations in Bacteroides diversity are crucial in the mitigation of DSS‐induced colitis by DHA. The principal co‐ordinate analysis (PCoA) employed to quantify beta diversity^[^ ^34^ ^]^ demonstrated that DHA therapy dramatically affected the gut microbiota composition in colitis animals (Figure 6H). To further identify specific taxonomic units with differential abundance between DHA‐treated and untreated colitis mice, we employed Linear discriminant analysis Effect Size (LEfSe) analysis to compare bacterial abundances at each taxonomic level.^[^ ^35^ ^]^ The Linear Discriminant Analysis (LDA) scores revealed the dominating groupings and their influence at various taxonomic gradations, ranging from phylum to species. There was a significant difference in the abundance of 18 bacterial genera between DHA‐treated and untreated colitis mice, as shown by an LDA score[log10]>3.6 and a *P*‐value <0.05 (Figure 6I,J). At the genus level, Bacteroides exhibited a significant increase in the fecal samples of colitis mice, and Muribaculaceae showed a notable increase in the fecal samples of colitis mice treated with DHA.

To further investigate the regulatory mechanism of DHA on EGC heterogeneity mediated by microbial products, we pre‐incubated EGCs with LPS (5 µg mL^−1^) for 2 h before DHA treatment to observe the stimulatory effect of microbial products on EGCs (Figure 6K). Consistent with the results of 16S rRNA sequencing, we observed significant activation of LPS‐pre‐treated EGCs, manifested by markedly increased expression of the EGC activation marker c‐Fos^[^ ^36^ ^]^ (Figure 6K,L). TLR4, which mediates EGC homeostasis^[^ ^37^ ^]^ and is a pivotal receptor for interactions between microbes and their products with EGCs,^[^ ^7^, ^38^, ^39^ ^]^ is also significantly upregulated in LPS‐pre‐treated EGCs. LPS stimulation further enhances the upregulation of downstream molecules NF‐κB and inducible nitric oxide synthase (iNOS) through TLR4, which is significantly correlated with the upregulation of S100β and GFAP proteins.^[^ ^33^, ^40^, ^41^ ^]^ This is consistent with previous research findings that human EGCs respond to microbial dysbiosis through differential expression of Toll‐like receptors (TLRs) and production of nitric oxide (NO), with pathogens (rather than probiotics) significantly inducing overexpression of the S100β protein, and EGCs releasing NO.^[^ ^40^ ^]^ It is noteworthy that treatment with DHA significantly reversed the aforementioned conditions, further indicating that the regulation of EGC heterogeneity by DHA under inflammatory conditions is achieved through the modulation of microbial products.

## 3 Discussion

**Figure 7:**
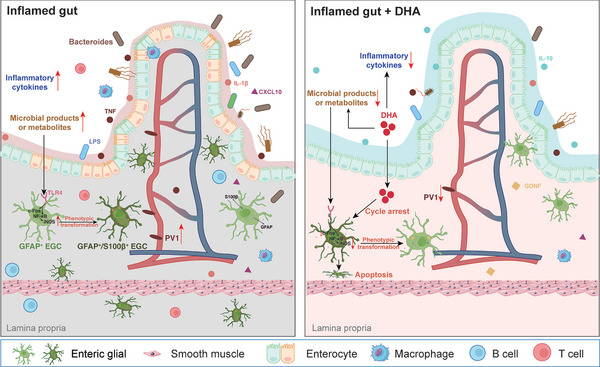
Graphical abstract: DHA regulates EGC phenotypic changes by promoting microbiota homeostasis to exert protective effects in colitis. In chronic colitis, microbial products or metabolites, along with inflammatory stimuli, can lead to concurrent damage to both the intestinal epithelial barrier (IEB) and the gut vascular barrier (GVB), accompanied by an increase in GFAP^+^/S100β^+^ enteric glial cells (EGCs) within the inflamed gut microenvironment. Treatment with dihydroartemisinin (DHA) effectively alleviates colitis and promotes the restoration of gut barrier function primarily through several mechanisms: DHA induces the phenotype conversion of GFAP^+^/S100β^+^ EGCs to GFAP^+^ EGCs or induces apoptosis of GFAP^+^/S100β^+^ EGCs while inhibiting the conversion of GFAP^+^ EGCs to GFAP^+^/S100β^+^ EGCs. The regulatory effect of DHA on EGC heterogeneity is mediated through the microbial products or metabolites/TLR4/NF‐κB/iNOS signaling axis, accompanied by the activation of c‐Fos. EGCs, enteric glial cells; DHA, dihydroartemisinin; LPS, lipopolysaccharide; GDNF, glial cell‐derived neurotrophic factor.

Although there is a substantial correlation between the pathophysiology of chronic enteritis and the phenotypic plasticity of EGCs,^[^ ^7^ ^]^ the underlying processes are still mostly unknown. In this study, we elucidated the pivotal role of EGC phenotypic alterations induced by changes in microbiota diversity during enteritis in regulating the integrity of the dual gut barrier and the severity of gut inflammation. We confirmed that DHA modulates the heterogeneity of EGCs by reshaping the gut microbiota homeostasis in colitis, thereby alleviating colitis progression. Specifically, DHA induces the transformation of GFAP^+^/S100β^+^ EGCs into GFAP^+^ EGCs or prevents the transition of GFAP^+^ EGCs into GFAP^+^/S100β^+^ EGCs while promoting apoptosis of GFAP^+^/S100β^+^ EGCs (**Figure** **7**).

Previous studies have reported the protective effects of DHA against IBD, primarily attributing this protective mechanism to DHA's regulatory effects on immune cells.^[^ ^42^, ^43^ ^]^ Yan et al. demonstrated that DHA protects mice from oxazolone (OXA) and 2,4,6‐trinitrobenzene sulfonic acid (TNBS)‐induced colitis by triggering apoptosis of activated CD4^+^ T cells, therefore regulating the balance of T helper (Th) and regulatory T (Treg) cells.^[^ ^42^ ^]^ Another artemisinin derivative, SM934, has been shown to exert protective effects against colitis by inhibiting neutrophils, macrophages, and the NF‐κB signaling pathway.^[^ ^43^ ^]^ The anti‐inflammatory effects of DHA are also evident in its alleviation of LPS‐induced neuroinflammation by inhibiting the PI3K/AKT pathway.^[^ ^44^ ^]^ In human and mouse models, Schneider et al. discovered a unique pathogenic P2X2‐dependent pathway that drives ATP‐induced enteric gliosis, inflammation, and disruptions in motility during intestinal inflammation.^[^ ^45^ ^]^ This prompts us to focus our attention on the ENS, specifically on EGCs.

Traditionally, EGCs have been considered supportive cells for enteric neurons. However, as our understanding of the diverse roles EGCs play in regulating gastrointestinal functions expands, this conventional perception of EGCs as passive cells is evolving. Due to their distribution across the gut wall's layers, EGCs can modify their activities in response to changes in their microenvironment. When they sense stimuli from their microenvironment, they can adjust the expression of their surface markers.^[^ ^25^ ^]^ As evidenced by this study, there was a significant increase in GFAP^+^/S100β^+^ EGCs during colitis, suggesting that local inflammatory changes in the microenvironment stimulate EGCs. As observed in Figure 4K,M, inflammation triggers an increase in the synthesis and secretion of IL‐1β by EGCs. Additionally, inflammation enhances the synthesis of CXCL10 in EGCs (Figure 4L) but impairs its secretion (Figure S4F, Supporting Information). Baidoo et al. found that aging in the human descending colon decreases the density of S100β^+^ EGCs in the myenteron, leading to colonic dysfunction.^[^ ^46^ ^]^ In addition to supporting neurons and the immune system, EGCs are reported to interact with epithelial cells to regulate gut permeability.^[^ ^25^ ^]^ Several studies have shown that EGCs can produce GDNF and S‐nitrosoglutathion (GSNO) to promote the expression of barrier‐related proteins, thereby enhancing the integrity of the IEB.^[^ ^47^, ^48^ ^]^ In our study, DHA treatment significantly enhances the ability of EGCs to synthesize GDNF, which may also be a crucial factor in the protective effect of DHA against colitis. Soret et al. suggested that supplementing exogenous GDNF helps alleviate dysbiosis caused by colitis, but this protective effect is ineffective against Bacteroides.^[^ ^49^ ^]^ In our study, DHA treatment did not increase EGC secretion of GDNF while alleviating the abnormally elevated proportion of Bacteroides in the feces of DSS‐induced colitis mice, indicating that the action of DHA on Bacteroides does not necessarily require mediation by GDNF. Undeniably, ablating EGCs in vivo leads to a sharp decrease in intestinal permeability.^[^ ^25^ ^]^ We believe that the effect of EGC ablation is not solely attributed to IEB damage but also extends to damage in the GVB. As observed in our study, colitis results in impairment of the dual intestinal barriers, concurrent with an increase in the proportion of GFAP^+^/S100β^+^ EGCs. This observation raises suspicions regarding the potential role of GFAP^+^/S100β^+^ EGCs in promoting the progression of intestinal inflammation.^[^ ^50^ ^]^

In Figures 4F and Figure S4D (Supporting Information), we observed an increase in the proportion of GFAP^+^ and S100β^+^ primary EGCs derived from mice that did not develop colitis but received DHA treatment. However, compared to the control group, there was no difference in the proportion of primary S100β^+^ EGCs derived from mice with colitis. Therefore, investigating the increase in the proportion of S100β^+^ EGCs induced by DHA administration would be meaningless based on this premise. Thus, we focus more on the significant increase in GFAP^+^/S100β^+^ EGCs and the remarkable decrease in GFAP^+^ EGCs observed in colitis mice. Besides, western blotting in vitro experiments are more indicative of phenotype changes in GFAP^+^/S100β^+^ EGCs (Figure 4H), as the EGCs treated in vitro are not influenced by other interfering factors present in the complex microenvironment in vivo while simultaneously exhibiting dual expression of GFAP and S100β. This consistency is demonstrated by the trend observed in both Figure 4G,H. When investigating the specific mechanism of DHA action on EGCs during colitis, RNA‐Seq analysis unveiled a noteworthy downregulation of genes in the DHA‐treated group compared to the untreated inflammatory group. These downregulated genes were notably enriched in two major KEGG pathways: cell growth and death, and replication and repair (Figure 5B). Subsequent mice and cell experiments provided further confirmation of these findings (Figure 5). To distinguish between normal and pathological signaling, glia only express more iNOS when there is inflammation.^[^ ^7^ ^]^ In the face of various stimuli within the gut microenvironment, EGCs often react rapidly, but they also tend to sacrifice themselves in the process. During clostridioides difficile infection, both clostridium difficile toxin B alone and in conjunction with pro‐inflammatory cytokines induce apoptosis in EGCs through the activation of three distinct signaling pathways mediated by caspases, calpains, and cathepsin B.^[^ ^51^ ^]^ TLRs, especially TLR4, as pattern recognition receptors (PRRs), are crucial in maintaining symbiosis between the gut microbiota and the host.^[^ ^52^ ^]^ Their expression in the enteric glial suggests the potential of the enteric glial lineage that directly responds to stimuli from the microbiota. As shown in Figure 6K, stimulation with LPS induces an increase in TLR4 expression in EGCs. At this point, EGCs are in an activated state, often referred to as reactive EGCs. “Reactive” describes the state in which EGCs respond to any degree of pathological or physiological disturbance. Reactive enteric gliosis is a highly complex and dynamic continuum characterized by environmental dependency and disease specificity, which can be considered as an attempt by EGCs to maintain internal balance.^[^ ^7^ ^]^

Research has shown that germ‐free mice or those treated with broad‐spectrum antibiotics, which lack microbiota, exhibit a significant reduction in EGCs in both the submucosal and myenteric plexuses.^[^ ^11^ ^]^ In mice with colitis, the more severe the extent of inflammation was associated with increased ratios of GFAP^+^/S100β^+^ EGCs, while concurrently, the proportion of Bacteroides increased in the gut microbiota, suggesting a possible correlation of GFAP^+^/S100β^+^ EGCs with Bacteroides in enteritis. Remarkably, treatment with DHA significantly reverses this phenomenon. S100β is known to play a role in regulating microbial diversity in the mouse intestine.^[^ ^33^, ^53^ ^]^ Our research suggests that GFAP, along with S100β, participates in this complex regulatory process simultaneously. Lei et al. investigated the effects of DHA on the gut microbiota of mice with acute colitis.^[^ ^54^ ^]^ In contrast to the reduced proportion of Bacteroides observed in the feces of acute colitis mice, we observed a significant increase in Bacteroides abundance in mice with chronic colitis (Figure 6E–G), suggesting that during chronic inflammation, Bacteroides may act as opportunistic pathogens contributing to the progression of colitis.

Overall, our study demonstrates that the damage to IEB/GVB occurring in chronic colitis is closely associated with the increase of GFAP^+^/S100β^+^ EGCs in inflamed colonic tissues. By suppressing the phenotypic change of GFAP^+^ EGCs to GFAP^+^/S100β^+^ EGCs, increasing the transition of GFAP^+^/S100β^+^ EGCs into GFAP^+^ EGCs, and encouraging the apoptosis of GFAP^+^/S100β^+^ EGCs, DHA slows the evolution of gut inflammation and enhances the function of the dual gut barrier. We confirmed that the regulatory effect of DHA on EGC heterogeneity is achieved through the modulation of intestinal microbiota homeostasis. DHA supplementation increased the abundance of gut microbiota in colitis mice, leading to an elevated proportion of beneficial bacteria (such as Muribaculaceae) and normalization of the proportion of opportunistic pathogens (such as Bacteroides), thereby reducing the stimulating effects of harmful microbes and their metabolites on EGCs.

To sum up, our work sheds light on the specific mechanisms underlying the progression of chronic colitis from the perspective of the ENS, aiding in the identification of innovative therapeutic targets for intervention in IBD and providing new insights for future strategies using DHA for IBD therapy. Ultimately, our findings aim to enhance the quality of life for IBD patients.

## 4 Experimental Section

### Animals

All mice utilized in this investigation shared a C57BL/6 background. Wild‐type male C57BL/6 mice, aged 6–8 weeks, were acquired from Beijing Vital River Laboratory Animal Technology Company in China. Ethical standards and animal welfare were upheld through approved protocols following the guidelines of the Animal Ethics Committee of Zhongnan Hospital of Wuhan University, China (Approval No. WP20230028). All experimental procedures adhered to the National Law on the Use of Experimental Animals in China.

### DSS‐Induced Colitis

Male wild‐type C57BL/6 mice were randomly assigned to control and colitis groups. All mice were housed in a 12‐hour light‐dark cycle specific pathogen‐free (SPF) environment, fed a standard diet, and acclimatized for one week before commencing the modeling procedure. There were five mice in each group. The colitis group received drinking water containing 2% DSS (molecular mass: 36000–50000 Da; MP Biomedicals, California, USA) for 7 days, followed by 7 days of distilled water, constituting one cycle. This cycle was repeated four times to establish a DSS‐induced chronic colitis model, effectively simulating the clinical process of remission and exacerbation in UC. The control group received distilled water throughout the experiment.

### Drug Administration

For the DHA treatment group, DHA (Aladdin Biochemical Technology Co., Ltd., China) was administered via intraperitoneal injection, consistent with the methodology outlined in prior studies by Zeng et al.^[^ ^55^ ^]^ The concentration of DHA was 20 mg kg^−1^ (body weight), diluted in corn oil (HY‐Y1888, MedChemExpress, USA). DHA administration occurred during the interlude period of the modeling procedure, precisely during the 7‐day interval of distilled water intake following the 7‐day DSS exposure. Refer to Figure 3A for the detailed modeling process.^[^ ^56^, ^57^ ^]^ Three separate observers calculated the disease activity index (DAI) of mice by daily monitoring of body weight, stool consistency, and occult blood.^[^ ^58^ ^]^ Body weight loss was quantified on a 0 to 4 scale (0, no change; 1, <5%; 2, 5–10%; 3, 11–20%; 4, >20%). Stool consistency was scored on a 0 to 4 scale (0, normal; 1, soft but formed; 2, extremely soft; 3, half diarrhea; 4, diarrhea). Fecal bleeding severity was assessed on a 0 to 4 scale (0‐2, none; 3, blood tracked in visible stool; 4, entirely rectal bleeding).^[^ ^59^ ^]^ Each mouse's spleen index was determined using the following formula: (Spleen Weight (g)/Body Weight (g)) × 100%.

On the 57th day, euthanasia was performed on the mice, followed by the removal and measurement of the length of the entire large intestine (cecum, colon, and rectum). The spleen was collected and weighed, and the spleen index (SI) was calculated. Visceral organs such as the heart, liver, spleen, lungs, and kidneys were collected, fixed overnight in 4% paraformaldehyde, embedded in paraffin, and sectioned into 4µm‐thick slices for subsequent staining with hematoxylin and eosin (H&E), as well as for further immunohistochemical or immunofluorescent staining. The leftover tissues were immediately liquid nitrogen flash‐frozen and kept at −80 °C.

### H&E Staining, Immunohistochemistry (IHC), and Immunofluorescence (IF) Assay

The conventional protocol for staining tissue slides with H&E was followed. The slides underwent two consecutive 15‐min xylene washes, and then five minutes of ethanol washes at progressively lower concentrations (100%, 100%, 95%, and 70%). Lastly, the slides were incubated for five minutes in deionized water. Hematoxylin and eosin were used to stain the prepared slides. Using a double‐blind methodology, two gastrointestinal pathologists assessed and scored changes in the intestinal tissues’ histopathology based on the following criteria: inflammation (severity and extent), epithelial alterations (erosion/ulceration), submucosal edema, crypt alterations (crypt loss, cryptitis/crypt abscess, regeneration/hyperplasia, and goblet cell loss), and percentage of pathology‐affected tissue area compared to the total tissue area on the slide. They employed a 0–4 scoring system: 0, inside standard deviations; 1, minimum; 2, gentle; 3, moderate; 4, severe.

For IHC, the dewaxed sections underwent high‐temperature (95–99°C) antigen retrieval in a 10 mmol L^−1^ citrate buffer (pH 6.0) for 10–15 min and were incubated with 3% hydrogen peroxide (H_2_O_2_) for 30 min to quench endogenous peroxidase activity. Then, block the slides with 5% goat serum for 30 min. Following this, the slides were incubated overnight at 4 °C in the specified concentration of the primary antibody. The next day, the slides were incubated for 1 h at 37 °C with the horseradish peroxidase (HRP)‐conjugated secondary antibody (Proteintech, PR30009). Finally, the detection was performed using the DAB (3,3′‐Diaminobenzidine) substrate kit (ZSGB‐BIO, ZLI‐9018, Beijing, China). The slides were protected and mounted with neutral resins after nuclei were counterstained with hematoxylin (Beyotime, Cat#C0105, China). We used an Olympus microscope to take light pictures of the stained slices.

For IF assay, the sections, having undergone antigen retrieval, were incubated in 5% goat serum at 37 °C for 1 h. Tissues were then incubated overnight at 4 °C with the designated primary antibody at an appropriate concentration. On the following day, tissue slices were incubated with the corresponding fluorescent secondary antibodies (1:400, Goat anti‐mouse, DyLight 549, A23310; and 1:300, Goat anti‐rabbit, DyLight 488, A23220; Abbkine, Wuhan, China) (1:300, Goat anti‐mouse, Alexa Fluor 488, 33106ES60; and 1:300, Goat anti‐rabbit, Alexa Fluor 647, 33113ES60, Yeasen Biotechnology, Shanghai, China) at 37 °C for 1 h. Following incubation with 4′,6′‐diamidino‐2‐phenylindole (DAPI) for 5–10 minutes, mounting was performed. Intervals between each step were washed with phosphate buffered saline (PBS). Finally, we used a Leica SP8 confocal fluorescence microscope to analyze the slides. Images were processed with Fiji imagej software (https://imagej.net).

### RNA Extraction and RT–qPCR Analysis

Total RNA was extracted from mouse colonic tissues using the RNA preparation kit (TRIpure Reagent, Aidlab, RN01, Beijing, China). The cDNA synthesis was performed with the ReverTra Ace qPCR RT Kit (Toyobo, FSQ‐101, Osaka, Japan). Quantitative real‐time polymerase chain reaction (qRT‐PCR) was conducted in a 10 µL reaction system using UltraSYBR Mixture (CWBIO, Jiangsu, China) on the Roche Light Cycler 96 (Roche, Basel, Switzerland). 2^(−ΔΔCt) was used to compute the relative mRNA levels, with GAPDH as the internal reference. Table S1 (Supporting Information) lists the precise primers used in the RT‐PCR.

### TEM Analysis

We employed TEM to study changes in colon ultrastructure in each group. Colon tissues were cut into cubic millimeters and immediately fixed in 2.5% glutaraldehyde (volume fraction) for 4 h at −4 °C. The samples were washed three times in PBS and fixed in 1% (volume fraction) of sodium tetroxide for 2 h. After drying in an ethanol gradient and embedding in epoxy resin, we used a diamond knife on an ultramicrotome (Leica, EM UC7, Germany) to cut ultrathin sections (60‐70 nm). The tissue was treated with 2% (volume fraction) uranyl acetate for 20 min. Finally, the materials were examined and photographed using a transmission electron microscope (HITACHI, HT7800, Japan).

### GVB Permeability Assessment (Gut Transit Assay)

24 hours before anesthesia with 2, 2, 2‐Tribromoethanol (Aibei Biotechnology, M2920, Nanjing, China), mice were subjected to a fasting period with free access to water. Following midline laparotomy, a 4 cm intestinal loop was exteriorized and ligated. Subsequently, 2 mg of 70 kDa FITC‐Dextran (FD70, Sigma‐Aldrich) was dissolved in 2 ml of sterile saline solution (0.9% NaCl) and intraluminally injected through the ligated intestinal segment.^[^ ^5^ ^]^ 1 h later, blood was collected from the heart, and the concentration of FITC‐dextran in plasma was measured using a fluorescence microplate reader (BioTek Epoch, USA). To achieve accurate quantification, the fluorescence intensity was adjusted with FITC‐glucan standards, as per the manufacturer's instructions. Concurrently, liver and spleen tissues from the mice were fixed with 4% paraformaldehyde for subsequent preparation of frozen sections (20 µm). The fluorescence density of FD70 in the liver and spleen was quantified using the Leica SP8 confocal fluorescence microscope.

### Cell Culture and Treatment

The primary umbilical vein endothelial cells (HUVEC) (PCS‐100‐013, ATCC) and rat enteroglial cell line EGC (CRL‐2690, ATCC) were grown in Dulbecco's Modified Eagle's Medium (DMEM, Hyclone), which included 10% heat‐inactivated fetal bovine serum (FBS, HyClone) and penicillin‐streptomycin (PS, HyClone). We pre‐incubated CRL‐2690 cells with Recombinant Rat TNF (PeproTech, 400–14, USA) for 24 h or LPS (Sigma‐Aldrich, USA) for 2 h to activate EGCs for subsequent experiments. The HT‐29 (HTB‐38, ATCC) human colonic epithelial cell line was grown in Roswell Park Memorial Institute (RPMI, HyClone) 1640 medium supplemented with 10% heat‐inactivated FBS (HyClone) and PS (HyClone). All cells were cultivated in a monolayer at 37 °C in humidified air containing 5% CO2.

### Flow Cytometry

EGC taken from the mouse colon's submucosa and lamina propria were centrifuged at 400 ×g for 5 minutes and washed once with PBS. The cells were then resuspended in Fixation/Permeabilization solution (BD Pharmingen, 554722, USA) and incubated at 4°C for 30 min. Subsequently, the cells were washed once with Perm/Wash™ buffer (BD Pharmingen, 554723, USA) and incubated in Perm/Wash™ buffer containing the appropriate concentration of fluorescent antibodies at 4°C in the dark for 1 h. The cells were washed and resuspended in the staining buffer (BD Pharmingen, 1029962, USA) for further flow cytometry analysis.

### Isolation of EGCs from the Colonic Submucosa and Lamina Propria of the Mice

Following the experimental procedure outlined by Wang et al.,^[^ ^26^ ^]^ EGCs were extracted from mouse colonic tissues. The mouse was euthanized and dissected, with the whole colon separated and the mesentery removed. The colon was then rinsed of residual fecal contents in pre‐cooled Dulbecco's phosphate buffered saline (DPBS). The colon was segmented into 3 cm sections, and, following the method described by Sundaresan et al.,^[^ ^60^ ^]^ the longitudinal muscle layer and the longitudinal muscle/myenteric plexus (LMMP) were sequentially removed. Subsequently, the colon segments were minced and incubated in the pre‐cooled sterile EDTA/HEPES/DPBS solution, followed by grinding to eliminate the mucosa. The suspension was purified through a 100 µm nylon cell strainer. The tissue was incubated in the commercially available cell recovery solution (Corning, 354253, USA) for 30 min, followed by a 10‐min grinding step to separate EGC from the lamina propria. The resulting mixture was filtered through a 40 µm strainer, centrifuged at 2000 × g for 5 min, and the sediment was collected to collect EGC, and analysis was performed using FlowJo analysis software (Treestar Inc., Ashland, OR, USA). EGCs were stained with the following antibodies: PE mouse anti‐GFAP, BD Pharmingen, 561483; S100β Alexa Fluor® 488, Santa Cruz, sc‐393919AF488.

### Enzyme‐Linked Immunosorbent Assay (ELISA)

Following the manufacturer's instructions (R&D Systems, Minneapolis, Minnesota), the levels of IL‐1β, TNF, and IL‐10 in mouse colonic tissues were measured using ELISA kits. Equal quantities of tissue samples from each group were homogenized in the PBS supplemented with a protease inhibitor mixture, followed by homogenization on ice using ultrasonication. The samples were centrifuged at 1000 × g for 15 min, and the supernatant was collected to measure the absorbance at 450 nm using a microplate reader (BioTek Epoch, USA). The concentrations of the corresponding cytokines in the samples were calculated based on a standard curve. LPS concentrations in mice's peripheral blood were measured using a kit (R&D Systems, Minneapolis, Minnesota) according to the manufacturer's instructions. According to the manufacturer's instructions, levels of GDNF (Elabscience, Wuhan, China), CXCL10 (Elabscience, Wuhan, China), and IL‐1β (R&D Systems, Minneapolis, Minnesota) in both intracellular and supernatant of EGCs were measured using ELISA kits.

### Western Blot Analysis

Total protein was extracted from the colonic mucosal layer using RIPA buffer containing a phosphatase and protease inhibitor mixture. Protein quantification was performed using a bicinchoninic acid (BCA) protein assay kit (Beyotime Biotech, P0009, China). Equal amounts of total protein were dissolved in 1× sodium dodecyl sulfate (SDS) sample buffer and denatured. Subsequently, proteins were separated by 7%−12% SDS‐polyacrylamide gel electrophoresis (PAGE) and transferred onto PVDF membranes (Millipore, IPVH00010, USA) on ice. After blocking with 5% bovine serum albumin (BSA), the membranes were incubated overnight at 4 °C with the corresponding primary antibodies: rabbit anti‐GAPDH (Proteintech, 10494‐1‐AP), mouse anti‐GFAP (Cell signaling, 3670), rabbit anti‐S100β (Cell signaling, 90393), rabbit anti‐Occludin (Proteintech, 27260‐1‐AP), rabbit anti‐Claudin‐1 (Proteintech, 28674‐1‐AP), rabbit anti‐Claudin‐2 (Proteintech, 26912‐1‐AP), rabbit anti‐PV1 (Cell signaling, 38238), rabbit anti‐ZO‐1 (Absin, abs122482, Shanghai, China), rabbit anti‐β‐Catenin (Proteintech, 51067‐2‐AP), rabbit anti‐Cleaved Caspase‐3 (Cell signaling, 9661), mouse anti‐Bcl‐2 (Proteintech, 68103‐1‐Ig), rabbit anti‐Bax (Proteintech, 50599‐2‐Ig), mouse anti‐c‐Fos (Abcam, ab208942), rabbit anti‐iNOS (Proteintech, 22226‐1‐AP), rabbit anti‐ NF‐κB p65 (Cell signaling, 8242), rabbit anti‐Phospho‐NF‐κB p65 (Cell signaling, 3033), mouse anti‐TLR4 (Proteintech, 66350‐1‐Ig). Following three washes in 1×TBST, the membrane was incubated with HRP‐conjugated secondary antibodies (1/4000, Proteintech), and visualized using chemiluminescence by a detection kit (Affinity, Shanghai). Membranes were imaged using a GeneGnome XRQ system, and band intensity was calculated using Fiji software.

### Human Patient Tissues with IBD

Human colon biopsies were obtained from the Department of Gastroenterology, Zhongnan Hospital of Wuhan University, China. IBD was diagnosed based on endoscopic and histological examination of the biopsy tissue. All human biopsies were collected with informed permission, and the Ethics Committee at Zhongnan Hospital of Wuhan University (Approval No. 2024062K) authorized the study. Colon biopsies with no abnormalities from healthy individuals of the same age and gender, such as those having screening colonoscopies, were utilized as controls.

### RNA‐Seq and Analysis

Total RNA was purified for mRNA using poly‐T oligo‐attached magnetic beads, followed by cDNA synthesis, blunt‐end conversion, adaptor ligation, and PCR. Sequencing on an Illumina Novaseq platform generated 150 bp paired‐end reads. Raw fastq data underwent fastp processing for high‐quality clean reads. A Hisat2 v2.0.5 index facilitated alignment, and StringTie (v1.3.3b) assembled mapped reads. featureCounts v1.5.0‐p3 quantified gene reads, and FPKM (fragments per kilobase of transcript per million mapped reads) values were calculated. DESeq2 analyzed conditions with biological replicates, while edgeR assessed conditions without replicates, setting a threshold of corrected P‐value 0.05 and an absolute fold change of 2.

Gene enrichment and functional annotation analyses of DEGs were carried out using either DAVID (the database for annotation, visualization and integrated discovery) Bioinformatics Resources or the GOtats R package, which was adjusted for gene‐length bias. Significantly enriched GO terms by DEGs were defined as those with an adjusted p‐value of less than 0.05. To examine the statistical enrichment of differential expression of genes in KEGG pathways, we utilized the R package ClusterProfiler. Heatmaps were created using the pheatmap package.

### Analysis of scRNA‐Seq Data

The pre‐aligned single‐cell transcriptomes of humans are sourced from the Gene Expression Omnibus dataset GSE114374, comprising the following samples: Human_HC_1: GSM3140593; Human_HC_2: GSM3140594; Human_UC_1: GSM3140595; and Human_UC_2: GSM3140596. The human mesenchyme was analyzed using an in‐house pipeline.^[^ ^61^ ^]^ To put it succinctly, cells expressing 200 or more genes, genes with nonzero values, and genes linked to at least 5 cells were chosen, and the library size and number of genes were submitted to strict quality control filters. Both datasets were subjected to a relaxed mitochondrial filter, which is two times the median absolute deviation of the fraction of mitochondrial genes as the median of the entire dataset. The Seurat R package^[^ ^62^ ^]^ was then used to read the filtered and normalized data for all upcoming analyses. Following data scaling and principal component analysis based on all genes, a Shared nearest neighbor (SNN) graph was created. This graph was then utilized in conjunction with an SNN modularity optimized clustering algorithm to determine cell groupings. Uniform Manifold Approximation and Projection (UMAP)^[^ ^63^ ^]^ graphs were used to depict the cells. To establish clusters, up to 1000 top genes (*P* < 0.05 and log fold change > 0.25) were chosen from among the DEGs in each cluster, which were found using the Wilcox test.

### Statistical Analysis

GraphPad Prism 9.5.1 (Graph Pad Software, San Diego, CA) was used for all statistical analysis and charting of results. When data fell under a normal distribution, the student's t‐test and analysis of variance were used appropriately. A value of *P* < 0.05 was considered statistically significant. These error bars represent the standard error of the mean (SEM) as measured.

## Conflict of Interest

The authors declare no conflict of interest.

## Author Contributions

P.Q., Y.C., and Q.Z. were associated with concept study and design. P.Q., L.L., X.C., H.N, C.X., and Y.H. performed experiments and data analysis. P.Q., Y.C., and H.W. wrote the final manuscript. P.Q. and S.W. performed visualization. Q.Z., M.Z., and Y.C. provided financial or technical support.

## Supporting information

Supporting Information

## Data Availability

The data that support the findings of this study are available from the corresponding author upon reasonable request.
