# Supplementary material for: Dihydroartemisinin Modulates Enteric Glial Cell Heterogeneity to Alleviate Colitis
Source: Adv Sci (Weinh). 2024 Jul 11;11(35):2403461. doi: 10.1002/advs.202403461 (PMC11425232; doi:10.1002/advs.202403461)
Supplement: Supplementary file 1 — Supporting Information [file ADVS-11-2403461-s001.docx]

**Supporting Information**

**Table S1.** **List of real-time qPCR primer sets (mouse)**

| **Gene** | **Forward primer (5ʹ-3ʹ)** | **Reverse primer(5ʹ-3ʹ)** |
| --- | --- | --- |
| IL1B | CAACGACAAAATACCTGTGG | AAACTCCACTTTGCTCTTGA |
| IL6 | GACTTCACAGAGGATACCAC | TCTGGCTTTGTCTTTCTTGT |
| IL10 | CTCTTACTGACTGGCATGAG | TAAGGAGTCGGTTAGCAGTA |
| IFNG | AAGTGGCATAGATGTGGAAG | CTGTTGCTGAAGAAGGTAGT |
| TNF | TATGTCTCAGCCTCTTCTCA | ATTTGGGAACTTCTCATCCC |
| GAPDH | TAACATCAAATGGGGTGAGG | TCATACTTGGCAGGTTTCTC |

qPCR, quantitative polymerase chain reaction; IL1B, interleukin 1 beta; IL6, interleukin 6; IL10, interleukin 10; IFNG, interferon gamma; TNF, tumor necrosis factor; GAPDH, glyceraldehyde 3-phosphate dehydrogenase.

**
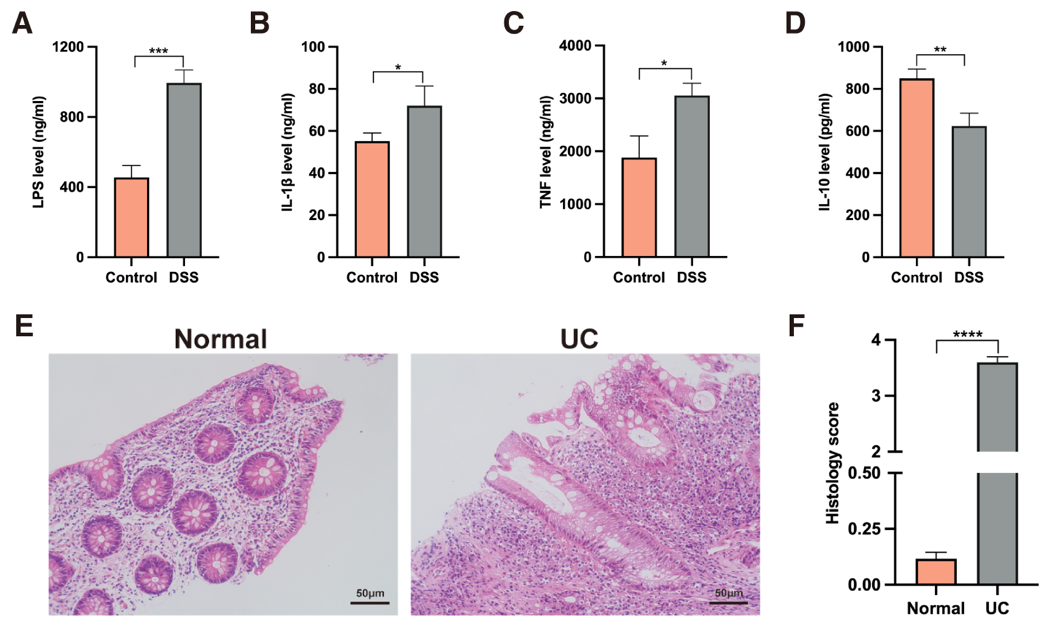
**

**Figure S1. Gut inflammation damage in enteritis**

(A) LPS levels in the peripheral blood of control and chronic DSS-induced colitis determined by ELISA. IL-1β (B), TNF (C), and IL-10 (D) levels in colonic tissue of control and chronic DSS-induced colitis determined by ELISA. (E) Representative images of colon tissue stained with hematoxylin and eosin (H&E) in healthy individuals and UC patients. (F) Statistics of histological score. LPS, lipopolysaccharide; DSS, dextran sulfate sodium; ELISA, enzyme-linked immunosorbent assay; UC, ulcerative colitis. Data are mean ± SEM. **P* <0.05, ***P* <0.01, ****P* <0.001, *****P* <0.0001.


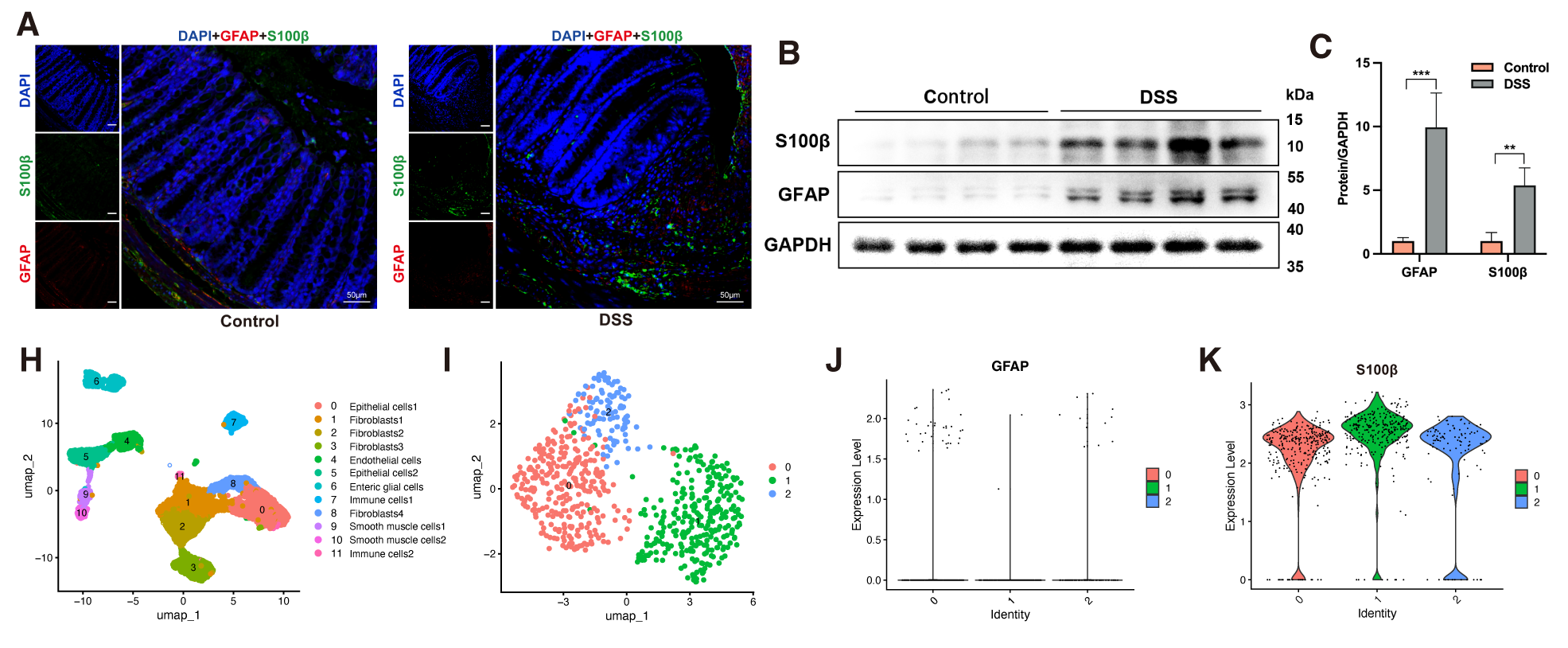
**Figure S2. Colitis induces remodeling of EGCs heterogeneity**

(A) Representative immunofluorescence images of EGCs labeled with GFAP (red) and S100β (green) in full-layer colonic sections of control and chronic DSS-induced colitis mice. DAPI (blue) stained the cell nuclei. (B-C) Western blot analysis of the expression of EGCs surface markers in the mucosa and lamina propria of control and chronic DSS-induced colitis mice. (H) Colonic mesenchymal cells from 2 healthy and 2 UC human patients were isolated and sequenced using 10× Chromium/hiSeq4000 instruments. The data were downloaded from the GEO database GSE114374. A UMAP of human colon mesenchymal cells is shown (n = 9281 cells). Descriptive cluster labels are displayed. Cluster 6 (n = 680), which expresses SOX10, GFAP, and S100β, is recognized as a population of human EGCs (hEGCs). (I) Subclustering analysis reveals three hEGC clusters (EGC#0–EGC#2). Violin plots of GFAP (J) and S100β (K) representing the distribution of individual EGC markers in human EGC cluster. DSS, dextran sulfate sodium; DAPI, 4’,6’-diamidino-2-phenylindole; UC, ulcerative colitis; GEO, Gene Expression Omnibus; GSE, GEO Series; UMAP, uniform manifold approximation and projection; EGCs, enteric glial cells. Scale bars are shown in the figures. n = 5 for each group. Data are mean ± SEM. ***P* <0.01, ****P* <0.001.

**
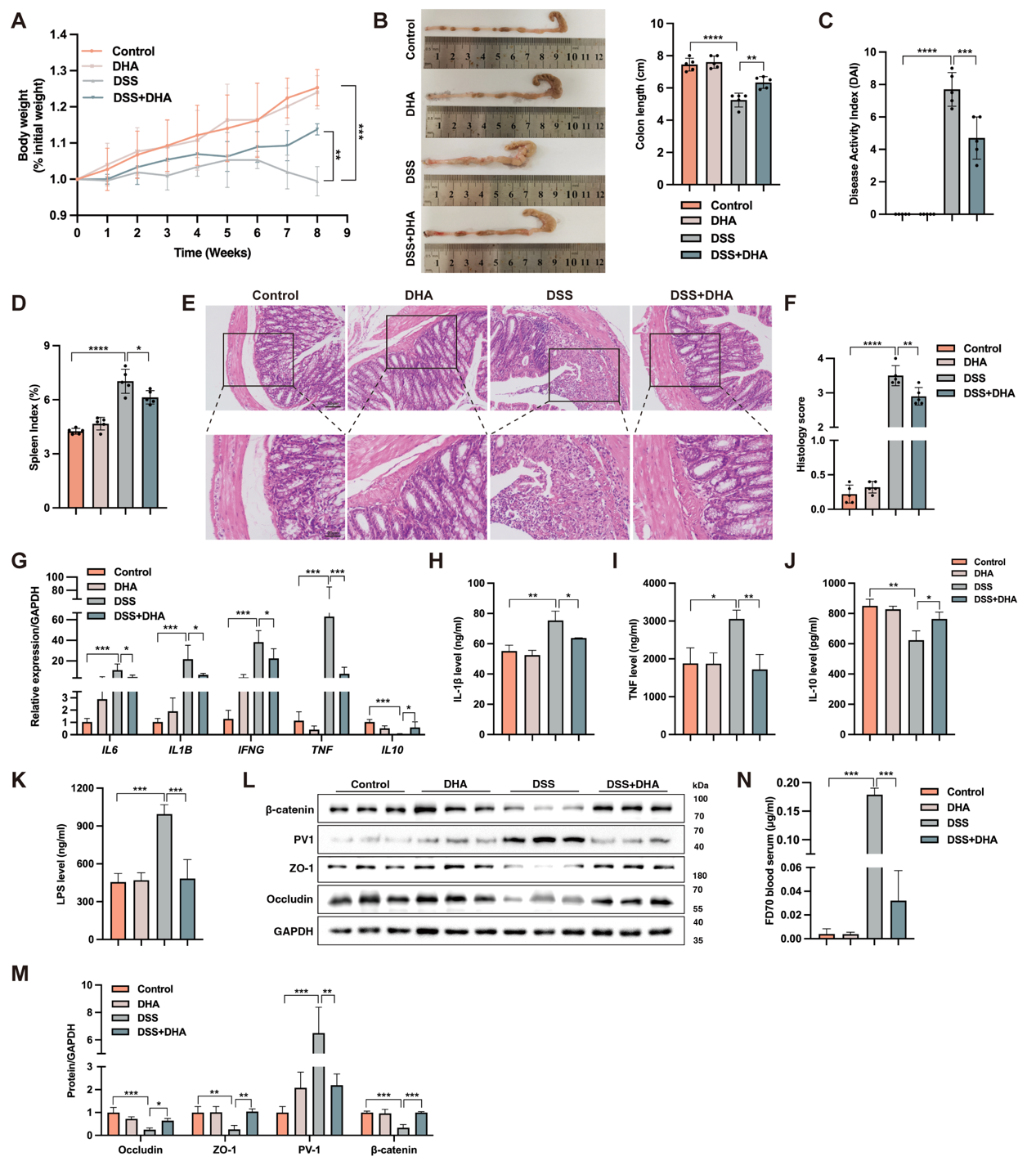
Figure S3. DHA alleviates gut inflammation in DSS-induced colitis**

Body weight (shown as the percentage of initial body weight) (A) and DAI (C) were monitored in colitis mice treated with or without DHA and corresponding control mice. (B) Representative images of the colon of each group. (D) Spleen index of each group. (E-F) Typical H&E-stained histological sections of each group. (G) qPCR analysis of inflammatory cytokine in the colon of colitis mice treated with or without DHA and corresponding control mice. IL-1β (H), TNF (I), and IL-10 (J) levels in colonic tissue of each group determined by ELISA. (K) LPS levels in the peripheral blood of each group determined by ELISA. (L-M) Western blot analysis of the level of IEB barrier proteins (ZO-1 and Occludin) and GVB barrier proteins (β-catenin and PV1) in the mucosa and lamina propria of colitis mice treated with or without DHA and corresponding control mice. (N) Serum FITC-dextran 70 kDa (FD70) concentration in control and chronic DSS-induced colitis mice treated with or without DHA. DHA, dihydroartemisinin; DSS, dextran sulfate sodium; DAI, disease activity index; LPS, lipopolysaccharide; IEB, intestinal epithelial barrier; GVB, gut vascular barrier; ELISA, enzyme-linked immunosorbent assay; PV1, plasmalemma vesicle associated protein. Scale bars are shown in the figures. n = 5 for each group. Data are mean ± SEM. **P* <0.05, ***P* <0.01, ****P* <0.001, *****P* <0.0001.

**
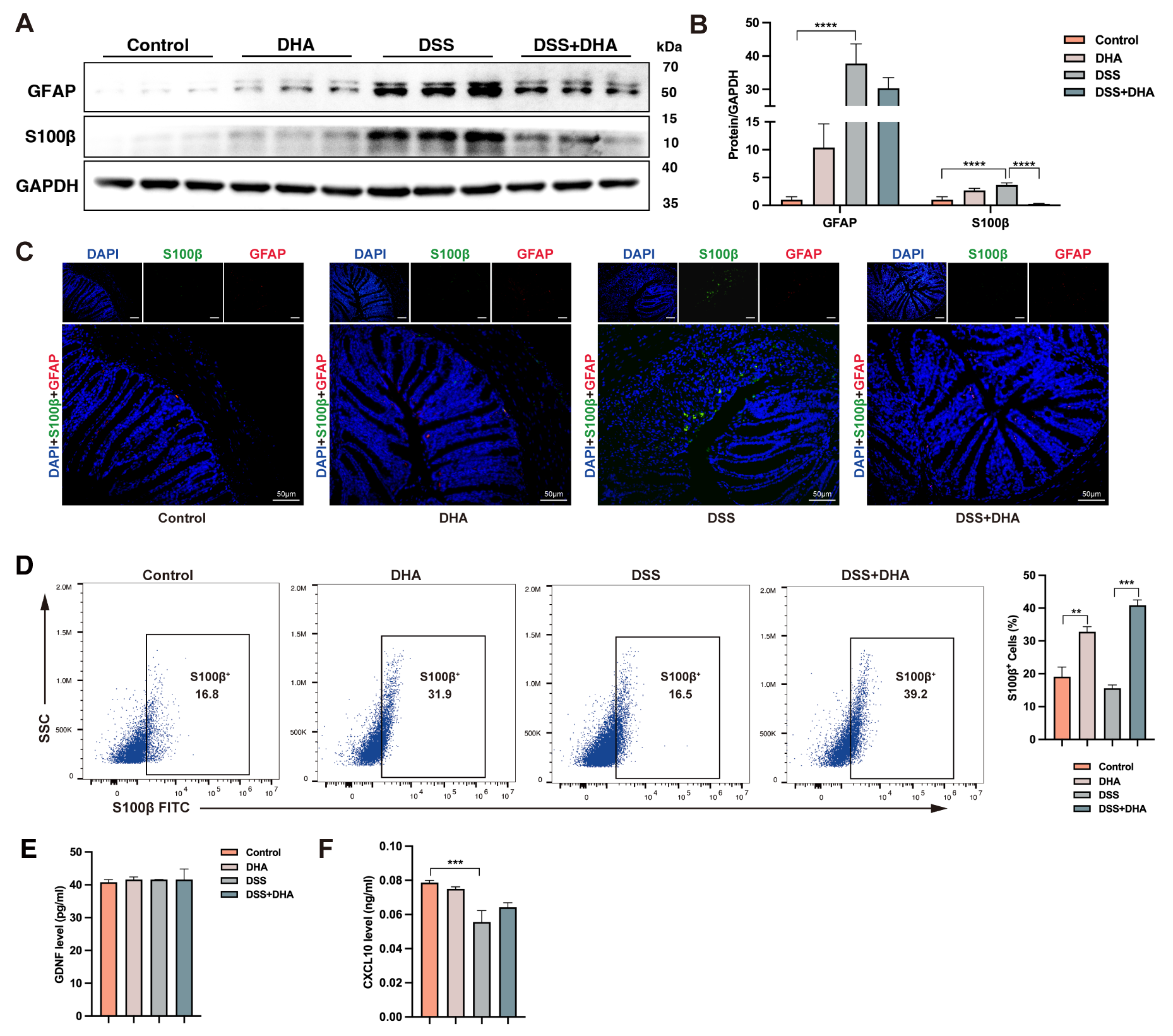
****Figure S4. DHA alleviates gut inflammation in DSS-induced colitis**

(A-B) Western blot analysis of the expression of EGCs surface markers (GFAP and S100β) in the mucosa and lamina propria of colitis mice treated with or without DHA and corresponding control mice. (C) Representative immunofluorescence images of EGCs labeled with GFAP (red) and S100β (green) in full-layer colonic sections of colitis mice treated with or without DHA and corresponding control mice. DAPI (blue) stained the cell nuclei. The percentage of S100β^+^ EGCs (D) were assayed in the colonic mucosa and lamina propria of colitis mice treated with or without DHA and corresponding control mice. The extracellular GDNF (E) and CXCL10 (F) levels in EGCs of each group determined by ELISA. DHA, dihydroartemisinin; DSS, dextran sulfate sodium; EGCs, enteric glial cells; DAPI, 4’,6’-diamidino-2-phenylindole; ELISA, enzyme-linked immunosorbent assay; GDNF, glial cell-derived neurotrophic factor; CXCL10, C-X-C motif chemokine ligand 10. Scale bars are shown in the figures. n = 5 for each group (A, C, and D). Data are mean ± SEM. ***P* <0.01, ****P* <0.001, *****P* <0.0001.

**
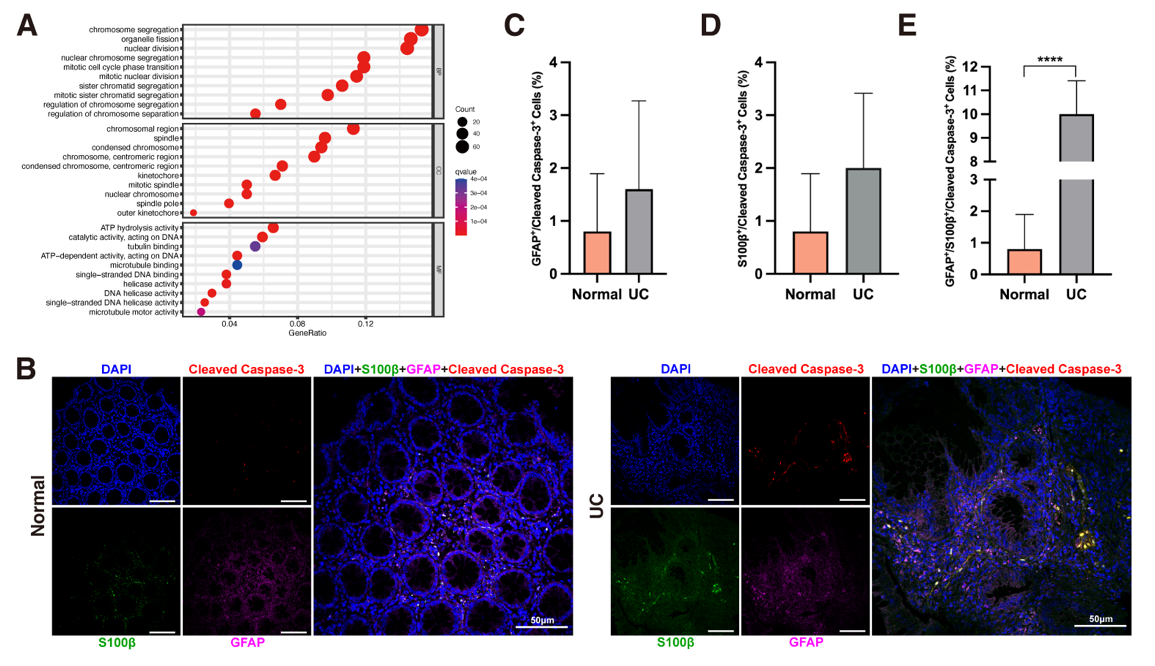
Figure S5. DHA induces apoptosis of GFAP^+^S100β^+^ EGCs**

(A) Top 10 results for GO annotations of differentially expressed genes (DEGs) in inflamed EGCs after DHA treatment. (B) Representative immunofluorescence images of Cleaved Caspase-3 (red) expression on EGCs stained with GFAP (magenta) and S100β (green) in the colonic biopsy of healthy individuals and UC patients. DAPI (blue) stained cell nuclei. Quantification of GFAP^+^/Cleaved Caspase-3^+^ cells (C), S100β^+^/Cleaved Caspase-3^+^ cells (D), and GFAP^+^/S100β^+^/Cleaved Caspase-3^+^ cells (E) in colon tissue from each group. About 50 cells were surveyed in each sample, and five fields of vision were used for quantification. GO, Gene Ontology; UC, ulcerative colitis; EGCs, enteric glial cells; DHA, dihydroartemisinin; DAPI, 4’,6’-diamidino-2-phenylindole. Scale bars are shown in the figures. Data are mean ± SEM. *****P* <0.0001.
